# Effects of γ-Aminobutyric Acid (GABA) Supplementation on Symptoms, Quality of Life, Intestinal Permeability, Systemic Inflammation and Gut Microbiota in Patients with IBS-D: A Randomized, Double Blind, Placebo-Controlled, Crossover Pilot Study

**DOI:** 10.3390/nu18101569

**Published:** 2026-05-14

**Authors:** Christian Lambiase, Lorenzo Cancelli, Riccardo Tedeschi, Antonio Grosso, Francesco Rettura, Rebecca Salemmo, Andrea Bottari, Fabio Filippini, Stefano Salvadori, Giulia Valdiserra, Letizia Campigli, Luca Antonioli, Matteo Fornai, Nicola de Bortoli, Massimo Bellini

**Affiliations:** 1 Gastrointestinal Unit, Department of Translational Research and New Technologies in Medicine and Surgery, University of Pisa, 56126 Pisa, Italy; 2 Regional Center for Functional and Motility Digestive Disorders, Azienda Ospedaliero-Universitaria Pisana, 56124 Pisa, Italy; 3 Gastroenterology Unit, Annunziata Hospital, Azienda Ospedaliera di Cosenza (AOCS), 87100 Cosenza, Italy; 4 Retrovirus Center, Department of Translational Research and New Technologies in Medicine and Surgery, University of Pisa, 56126 Pisa, Italy; 5 Institute of Clinical Physiology, National Research Council, 56124 Pisa, Italy; 6 Unit of Pharmacology and Pharmacovigilance, Department of Clinical and Experimental Medicine, University of Pisa, 56126 Pisa, Italymatteo.fornai@unipi.it (M.F.)

**Keywords:** GABA, γ-aminobutyric acid, irritable bowel syndrome, IBS

## Abstract

**Background/Objectives:** Recent studies have shown that GABA reduces visceral hypersensitivity and improves intestinal permeability in a post-inflammatory irritable bowel syndrome (IBS) rat model. We aimed to assess the efficacy of a GABA-based supplement in IBS patients with diarrhea (IBS-D), focusing on symptoms relief, quality-of-life improvement, mucosal barrier function, systemic microinflammation and gut microbiota. **Methods**: In this double-blind, placebo-controlled, crossover study, 20 IBS-D patients were randomized to receive GABA or placebo for two four-week treatment periods separated by a two-week washout. Efficacy was assessed using IBS Symptom Severity Score (IBS-SSS) and Short-Form Health Survey-36 (SF-36). Circulating levels of lipopolysaccharide-binding protein (LBP), Tumor Necrosis Factor-α (TNF-α) and interleukin (IL)-1β were measured before and after each treatment. **Results**: Eighteen patients completed the study. A clinical response (≥50-point reduction in IBS-SSS) was observed in 66.7% of patients during GABA treatment versus 33.3% with placebo. GABA produced a significant reduction in the IBS-SSS total score (*p* = 0.02) and in the bowel satisfaction item of the questionnaire (*p* = 0.02). Regarding quality of life, GABA significantly improved the “Emotional limitation” domain compared with placebo (*p* = 0.009). GABA treatment also led to a decrease in circulating LBP (*p* = 0.06) and IL-1β (*p* = 0.02) levels compared to placebo, although only the reduction in IL-1β reached statistical significance. In contrast, no substantial remodeling of the gut microbiota was observed. **Conclusions**: In this pilot study, GABA treatment led to a significant improvement in IBS-D symptoms compared with placebo and was also more effective in enhancing emotional wellbeing. GABA appeared to have a possible effect on intestinal permeability indirectly assessed through LBP, consistent with preclinical findings, and significantly reduced systemic inflammation. GABA may represent a promising therapeutic option for IBS, deserving further investigation in larger clinical trials.

## 1. Introduction

Irritable bowel syndrome (IBS) is a chronic disorder of gut–brain interaction characterized by recurrent abdominal pain associated with alterations in bowel habits, in the absence of an identifiable organic or inflammatory disease [1]. According to the Rome IV criteria, IBS can be subclassified based on the predominant stool pattern into diarrhea-predominant (IBS-D), constipation-predominant (IBS-C), mixed (IBS-M) and unclassified (IBS-U) subtypes [1]. IBS is highly prevalent worldwide, affecting approximately 4–10% of the general population [2,3].

IBS is associated with a substantial impairment in health-related quality of life [4]. Patients frequently report persistent symptoms, psychological comorbidities such as anxiety and depression, reduced work productivity, and dissatisfaction with available treatments [5,6,7,8]. Despite the broad range of therapeutic options currently recommended (including dietary interventions, probiotics, antispasmodics, antidiarrheals, bile acid sequestrants, gut–brain neuromodulators, and psychological therapies), treatment response remains heterogeneous and often incomplete, particularly regarding abdominal pain [9,10,11]. This unmet clinical need is especially evident in IBS-D patients, who often display more severe pain, increased intestinal permeability, and features of low-grade mucosal inflammation [2,11,12,13].

The limited efficacy of currently available treatments reflects the complex and multifactorial pathophysiology of IBS, which involves dysregulation of gastrointestinal motility, visceral nociception, immune activation, epithelial barrier function, gut microbiota composition, and bidirectional gut–brain communication within the microbiota–gut–brain axis [2,11,14]. Consequently, there is growing interest in therapeutic strategies targeting upstream mechanisms capable of modulating multiple pathophysiological components simultaneously.

Among these mechanisms, γ-aminobutyric acid (GABA)-mediated signaling has emerged as a biologically plausible target. GABA is the main inhibitory neurotransmitter in the central nervous system, but it also plays a relevant role at a peripheral level, including within the enteric nervous system, intestinal epithelium, immune cells, and gut microbiota [14,15,16,17,18]. Experimental and translational studies have shown that GABAergic signaling modulates visceral pain transmission, gastrointestinal motility, intestinal epithelial barrier impairment, oxidative stress and immune-inflammatory responses [18,19,20,21].

Evidence suggests that GABAergic dysregulation may be particularly relevant in IBS-D patients. Reduced circulating and mucosal GABA levels, together with altered expression of enzymes, transporters, and receptors involved in GABA signaling, have been reported in patients with IBS-D compared with healthy controls [22]. Such alterations may contribute to the impaired inhibitory control of nociceptive pathways (i.e., visceral hypersensitivity), as well as to epithelial barrier dysfunction and immune activation, which are recognized features of IBS-D pathophysiology [2,14].

Based on this background, interventions aimed at restoring or enhancing GABAergic signaling represent a promising therapeutic approach. In this regard, a combination of GABA and *Melissa officinalis* have been shown to interact with GABAergic pathways to exert anti-inflammatory and anti-nociceptive effects [23]. In a preclinical model of post-inflammatory IBS, a combined formulation of GABA and *Melissa officinalis* reduced visceral hypersensitivity, improved epithelial barrier integrity, attenuated oxidative stress, and modulated enteric and spinal glial activation (mechanisms closely linked to chronic abdominal pain and symptom persistence) [22,23].

However, despite a strong mechanistic rationale and encouraging preclinical evidence, the efficacy of GABA-*Melissa officinalis*-based product has not yet been evaluated in randomized clinical trials involving patients with IBS-D. To date, clinical evidence supporting GABA-targeted interventions in IBS remain limited, and high-quality trials are needed to determine whether modulation of GABAergic pathways translate into clinically meaningful benefits [15,22,24].

Therefore, the aim of the present randomized, double-blind, placebo-controlled, cross-over pilot clinical trial was to evaluate the efficacy of a GABA–*Melissa officinalis* formulation in patients with IBS-D in terms of IBS symptoms, quality of life and symptoms of anxiety and depression. Other aims include the indirect evaluation of the intestinal epithelial barrier, systemic low-grade inflammation, and intestinal microbiota.

## 2. Materials and Methods

### 2.1. Ethical Approval

Data were collected from subjects enrolled between April 2023 and March at the Gastrointestinal Unit of Azienda Ospedaliero-Universitaria Pisana. This study was conducted according to the Declaration of Helsinki and the Good Clinical Practice guidelines. It received institutional review board approval from the “Comitato Etico Area Vasta Nord-Ovest” (number 23485 of 2 February 2023) and was registered at ClinicalTrials.gov (NCT06755489). This clinical trial followed the CONSORT guidelines. All participants provided written informed consent before engagement with the study.

### 2.2. Participants and Inclusion/Exclusion Criteria

Subjects aged between 18 and 75 years old with a positive diagnosis of IBS-D (according to Rome IV criteria [1]) were enrolled in the study. The diagnosis was performed by skilled neurogastorenterologists in a tertiary center specialized in disorders of gut–brain interaction. A positive diagnosis of IBS-D was made according to Rome IV criteria [1].

Patients with other subtypes of IBS and/or with any relevant organic, systemic or metabolic disease or abnormal laboratory values considered clinically significant, or intestinal organic diseases (including celiac disease, food allergies or infective/inflammatory bowel diseases (Crohn’s disease, ulcerative colitis, infectious colitis, ischemic colitis, microscopic colitis)) were excluded from the study. Patients who underwent previous major abdominal surgery (excluding appendicectomy) and patients with active malignancy of any type or history of malignancy or recurrence in the last five years were also excluded. Furthermore, use of pre-probiotics, topical or systemic antibiotics and prokinetics within 15 days before treatment start represented an exclusion criterion. Pregnant or breastfeeding women, patients chronically using laxatives, and/or with recent history or suspicion of alcohol or drug abuse were also excluded.

### 2.3. Study Design and Randomization

**Figure 1:**
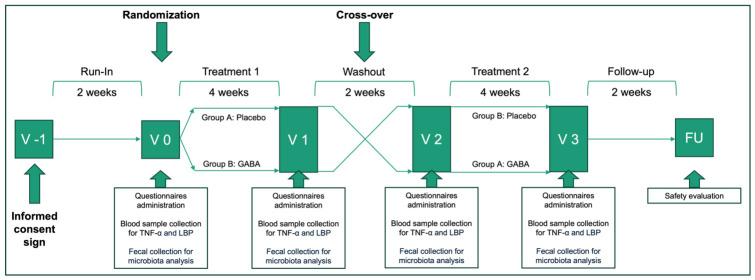
Study design.

This was a pilot, monocentric, randomized, double-blind, placebo-controlled, cross-over study. A schematic overview of the study is presented in Figure 1. Before randomization and after signing the informed consent form, each patient underwent a two-week run-in period.

After the run-in phase (visit 0, V0), patients were re-evaluated to confirm their eligibility for enrollment and, if eligibility was confirmed, were randomized in a 1:1 ratio to two possible treatment sequences (Group A: placebo followed by study supplement; Group B: study supplement followed by placebo) in a two-period cross-over design. Allocation to treatment sequence was balanced across participants. Both participants and investigators were blinded to treatment received and treatment sequence.

A washout period of two weeks was observed between the two treatment periods. The length of the washout was estimated on the half-life of GABA (24–48 h) and according to manufacturer’s indications.

Overall, the study had a length of 14 weeks for each patient. Clinical evaluations in presence at the center were scheduled at the beginning of the study (screening visit, V-1), at the beginning and ending of first treatment period (V0, V1) and second treatment period (V2, V3) (Figure 1). A follow-up telephonic evaluation was scheduled after 2 weeks from the end of the second treatment period for safety evaluations.

Empty medication packages were collected at V1 and V3 to verify compliance with treatment. Safety was assessed according to the occurrence of adverse events (frequency, intensity and relationship with treatment) and by vital signs. Adverse events were evaluated by the principal investigator for severity and whether they were likely to be attributable to the intervention.

### 2.4. Intervention and Data Collection

The intervention consisted of a 4-week intake of a commercially available food supplement in the form of a tablet containing 250 mg of gamma-aminobutyric acid (GABA) and 50 mg of a dry extract (ethanol/water) of *Melissa officinalis* (Depofarma, Treviso, Italy) [25,26,27].

Patients were instructed to take one tablet three times daily. The placebo consisted of a tablet that was indistinguishable from the intervention product in color, taste, and smell, but contained neither GABA nor *Melissa officinalis* dry extract.

At the beginning and at the end of each treatment periods, the following clinical questionnaires were administered to patients:

- Short-Form 36 (SF-36) [28]. It is a multiparametric tool designed to assess health-related quality of life in the general population. It consists of 36 items divided into eight domains, each investigating a different aspect of health. Each domain yields a score ranging from 0 to 100, where 0 represents the worst possible health status and 100 the best possible health status. Two summary indices can also be calculated: one for physical functioning (physical composite score, PCS) and one for mental functioning (mental composite score, MCS), standardized to a norm-based score of 50 ± 10 [28].
- Irritable bowel syndrome symptom severity score (IBS-SSS) [29]. It is a score calculated to assess the intensity of IBS symptoms. The score is based on five items: severity of abdominal pain, number of days with abdominal pain, severity of abdominal bloating/distention, dissatisfaction with bowel habits, and interference of IBS with daily life. Each item is scored on a visual analog scale ranging from 0 to 100. A total score of <75 points is considered indicative of normal status or remission, and the maximum possible total score is 500. Scores of 75–175 indicate mild IBS, scores of 176–300 indicate moderate IBS, and scores > 300 indicate severe IBS. Within this system, a “response” to therapy is defined as a reduction in the total IBS-SSS score of at least 50 points [29].
- Hospital anxiety and depression scale (HADS) [30]. It is a self-assessment scale frequently used to evaluate psychological distress in non-psychiatric patients. It includes 14 items divided into two subscales: 7 assessing anxiety symptoms (HADS-A) and 7 assessing depressive symptoms (HADS-D). Each item is scored from 0 to 3, with total scores for each subscale ranging from 0 to 21 [30].

Furthermore, information on daily bowel movements, stool consistency (assessed using the Bristol stool chart [BSC]) [31], daily presence and intensity of abdominal pain, adverse events and concomitant medications was recorded during each treatment period using a daily diary.

### 2.5. Evaluation of Plasma Levels of Lipopolysaccharide-Binding Protein (LBP), (IL)-1β and Tumor Necrosis Factor (TNF)

Lipopolysaccharide-binding protein (LBP), (IL)-1β and tumor necrosis factor (TNF) levels in plasma were measured by enzyme-linked immunosorbent assay (ELISA) kits (Human LBP ELISA Kit [ab213805, Abcam, Cambridge, UK], Human IL-1β DuoSet ELISA kit [DY201-05, R&D Systems, Minneapolis, MN, USA] and Human TNF-α ELISA Kit [BMS223-4, Invitrogen/Thermo Fisher Scientific, Waltham, MA, USA]) as described by the manufacturer’s instructions. For this purpose, blood samples collected at each time point were centrifuged for 10 min at 4000× *g*. Supernatants were collected and stored at −80 °C until analysis. Aliquots (100 μL) of plasma were used to perform the assay. The results were expressed as picogram per milliliter (ρg/mL).

### 2.6. Fecal Sample DNA Extraction and Metagenomic Sequencing

Fecal samples were collected before and after each treatment cycle (V0, V1, V2, V3). All samples were stored in sterile containers containing a preservative (DNA/RNA Shield™; Zymo Research, Irvine, CA, USA) and stored at −80 °C.

The extraction of microbial DNA from fecal samples was performed using the QIAamp^®^ PowerFecal^®^ Pro DNA Kit (QIAGEN, Hilden, Germany). This method combines a mechanical—with bead beating—and chemical methodology in order to ensure high purity and integrity of nucleic acids.

A DNA quantification for quality check was performed before the analysis using the Qubit™ dsDNA Broad Range Assay Kit Thermo Fisher Scientific, Waltham, MA, USA) and the Qubit™ 3 Fluorometer (Thermo Fisher Scientific, Waltham, MA, USA). It was ensured that all the DNA quantifications were adequate for NGS library preparation.

Next-generation sequencing (NGS) analysis was outsourced to Xenovea (Austria).

Library was prepared with KAPA HiFi HotStart Ready Mix Kit (Roche, Basilea, Switzerland) and Nextera XT v2 Index Kit (Illumina, San Diego, CA, USA). A 16S rRNA gene sequencing service was performed with NextSeq2000 (Illumina, San Diego, CA, USA).

### 2.7. Fecal Microbiota Bioinformatic Analysis

Paired-end 16S rRNA gene sequences were processed using QIIME 2 [32] (version 2024.10). Sequence denoising, quality filtering, and chimera removal were performed with DADA2 [33]. Forward and reverse reads were truncated at 300 base pairs, and no bases were trimmed from the 5′ ends. Taxonomic classification of representative sequences was performed using the QIIME 2 scikit-learn naive Bayes classifier trained on the SILVA 138 99% OTU database [34].

Alpha and beta diversity analyses were performed on the QIIME 2 profiles using the SciKit-bio (version 0.5.9), SciKit-learn (version 1.1.3), and SciPy (version 1.10.1) Python libraries. Multidimensional Scaling (MDS) on arcsine square root-transformed abundances was conducted using different distance metrics: Jaccard and Bray–Curtis. PERMANOVA and Mann–Whitney tests were performed using the SciKit-bio library. To identify taxa differentially abundant between treatments, MaAsLin (version 3) [35] was applied on raw taxonomic profiles. TSS normalization and LOG transformation were applied on the relative abundance data. Only microbial features above 10% prevalence were employed. Linear models were corrected by sex, age and BMI and corrected by abundance at baseline. For abundances at the V3, the wash period was considered as baseline. For the analysis of microbial functions, the relative abundance of the manually curated and associated microbial taxa were collapsed (summed up). Statistical analyses using MaAsLin for microbial functions were performed in a similar manner as with the taxa.

### 2.8. Quantification of GABA and Rosmarinic Acid in Tablets

To confirm the dose of GABA and rosmarinic acid in each tablet used in the study, HPLC analysis have been performed by an independent laboratory (LabAnalysis, Pavia, Italy). Unless otherwise stated, all solvents and chemicals were purchased from Sigma Aldrich/Merk (Darmstadt, Germany).

#### 2.8.1. GABA RP-HPLC

RP-HPLC analysis for gamma-aminobutyric acid (GABA) [36] was carried out using an HPLC system equipped with a UV detector (Agilent 1260 Infinity II, Agilent Technologies Inc, Santa Clara, CA, USA). Separation was performed at 30 °C using a Luna C18 column (250 mm × 4.6 mm, 5 μm, Phenomenex, Torrance, CA, USA). The gradient was performed between eluent A (methanol, LiChrosolv®) and eluent B (0.05 M sodium acetate and 1% THF, LiChrosolv®, in water): 0–2 min: 75% B; 20 min: 49% B; 50–55 min: 20% B; 56–60 min: 75% B. The flow rate was maintained at 1.0 mL/min, and the injection volume was 10 μL. The UV detector was set at 330 nm for both identification and quantification.

GABA was extracted from nutraceutical products using 0.1 N hydrochloric acid in an ultrasonic bath, followed by filtration through a 0.45 μm membrane filter and derivatization with ortho-phthalaldehyde (OPA) solution prior to analysis. A calibration curve was obtained at 330 nm between 2.50 and 50.00 μg/mL (RSD% < 20%).

#### 2.8.2. Rosmarinic Acid RP-HPLC

RP-HPLC analysis for rosmarinic acid, considered as the main active component [37,38,39,40,41,42], was based on the European Pharmacopeia monograph for *M. officinalis* [43] and was carried out using an HPLC system equipped with a UV detector (Agilent 1260 Infinity II, Agilent Technologies Inc, Santa Clara, CA, USA). Separation was performed at 30 °C using a Luna C18 column (250 mm × 4.6 mm, 5 μm, Phenomenex, Torrance, CA, USA). The gradient was performed between eluent A (0.5% phosphoric acid in water) and eluent B (acetonitrile, LiChrosolv®): 0–5 min: 95% A; 10 min: 90% A; 11–15 min: 80% A; 17–20 min: 70% A; 21–30 min: 95% A. The flow rate was maintained at 1.0 mL/min, and the injection volume was 10 μL. The UV detector was set at 330 nm for identification and quantification.

Rosmarinic acid was extracted from representative samples using a methanol:water (70:30) mixture in an ultrasonic bath for 30 min, followed by filtration through a 0.45 μm membrane filter. A calibration curve, using rosmarinic acid Reference Standard (cat. Y0000786, European Directorate for the Quality of Medicines) was obtained at 330 nm using a linear model through the origin between 0.50 and 10.00 μg/mL (RSD% 20%).

### 2.9. Statistical Analysis

#### 2.9.1. Sample Size Calculation

The sample size was calculated considering the study a randomized, double-blind, placebo controlled 2 × 2 crossover design. Given the prominent role of chronic gastrointestinal pain in patients with IBS-D, the “bodily pain” subdomain of the SF-36 was selected to calculate the sample size. This domain was considered the most clinically relevant measure of treatment effect in this population and IBS patients showed impairment in several SF-36 subdomains, including “bodily pain” [44].

A between-treatment difference of 20 points in the SF-36 Bodily Pain score (0–100 scale) was assumed to represent a clinically meaningful improvement. The standard deviation of the Bodily Pain domain was assumed to be 27.65 points, based on normative data from the Italian population [28]. A within-subject correlation of 0.5 between treatment periods was assumed.

Under these assumptions, with a two-sided significance level of 0.05 and 80% power, a total of 16 subjects were required to complete the crossover study (8 per treatment sequence). To account for potential dropouts, the target sample size was increased accordingly to 20 (20% dropout).

#### 2.9.2. Statistical Methods

Continuous variables were summarized using median and interquartile range (IQR), while categorical variables were reported as absolute and percentage values. Given the small sample size and the non-normal distribution of the outcome variables, statistical analyses were performed using the Aligned Rank Transform (ART) analysis of variance (ANOVA), a non-parametric approach appropriate for factorial designs that allows the evaluation of main effects and interactions. A potential carryover effect was explored by comparing the sum of the outcome values across the two periods between treatment sequences using a Wilcoxon rank-sum test.

Statistical significance was set at *p* < 0.05. All statistical analyses were performed using R (version 4.5.1) within RStudio (version 2026.01.0, Build 392) or GraphPad Prism 9.0 software (GraphPad Prism, San Diego, CA, USA).

## 3. Results

### 3.1. Patients’ Characteristics

**Figure 2:**
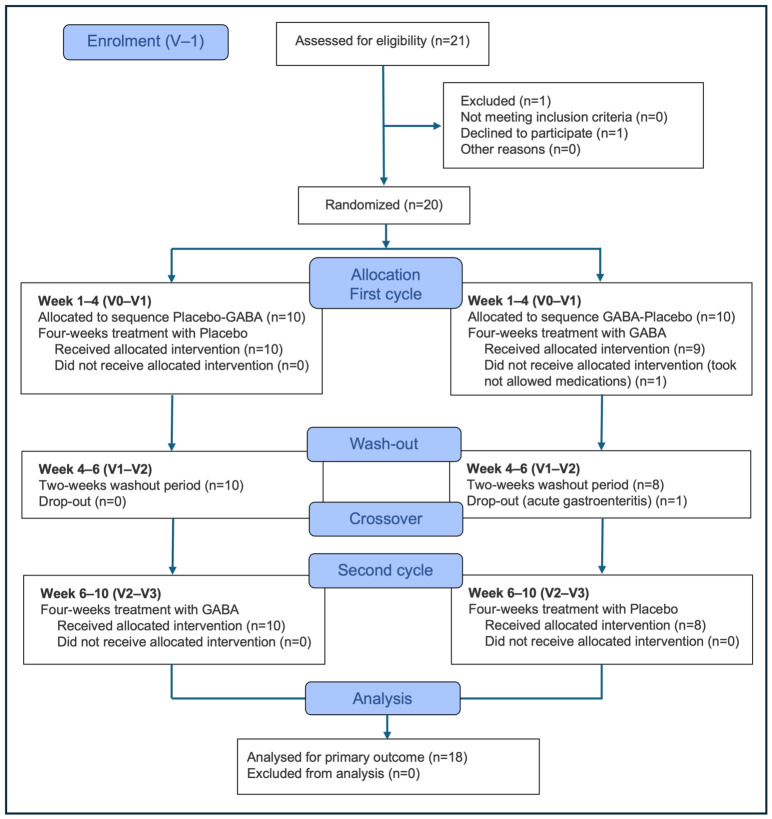
Study flow-chart.

Between April 2023 and March 2025, 21 patients were screened for inclusion (Figure 2). One patient refused to participate, and 20 patients were enrolled in the clinical trial and randomized, after a two-week run-in period.

Eighteen patients completed the study and were included in the analysis of primary and secondary outcomes. Patients’ demographic features are presented in Table 1. Ten females (55.6%) and eight males (44.4%), with a median age of 28.0 (11.8) years were included in the study. Group A arm (patients who took placebo at cycle 1 and GABA-*Melissa officinalis* at cycle 2) did not differ significantly regarding baseline characteristics from Group B (patients who took GABA-*Melissa officinalis* at cycle 1 and placebo at cycle 2), except for the sex (Table 1).

Fourteen patients (77.8%) had concomitant diseases at baseline: asthma (six cases), autoimmune hypothyroidism (four cases), appendicectomy (two cases), previous infective diseases (two cases), hemorrhoidectomy (two cases), chronic headache (one case), anxiety-depressive syndrome (one case), allergic rhinitis (one case), and lumbar discopathy (one case). Patients continued with their currently recommended chronic treatment for their comorbidities throughout the study (if compatible with inclusion and exclusion criteria).

### 3.2. Quality of Life

The impact of GABA on patients’ quality of life was assessed through the multidomain SF-36 questionnaire. During cycle 1, treatment with GABA was associated with a significantly greater improvement in the “Role Limitations—Emotional” domain compared with placebo (median change: 16.7 [83.3] vs. 0.0 [0.0]; *p* = 0.009, Table 2). No statistically significant differences between GABA-*Melissa officinalis* and placebo were observed for the other SF-36 domains, although numerical improvements were generally more pronounced during the GABA phase for “Role Limitations—Physical”, “Mental health”, “Social functioning” and “Bodily pain” domains of the score. No relevant carry-over effects were observed.

### 3.3. IBS Symptoms and Psychological Distress

**Figure 3:**
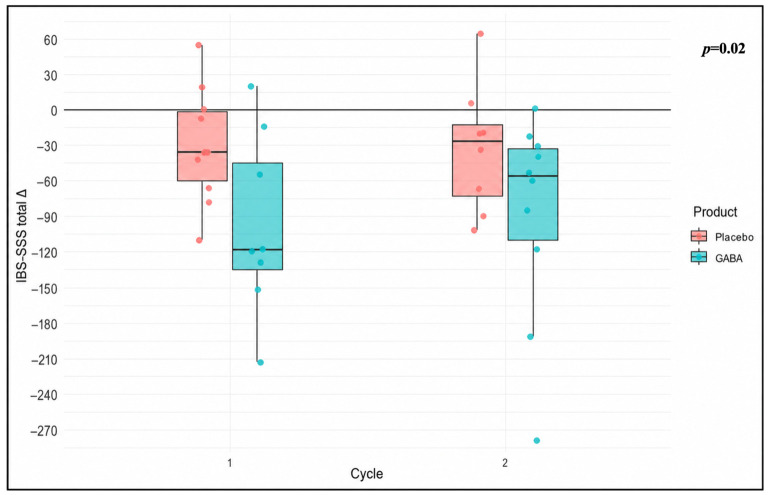
Difference in IBS-SSS total score according to product and cycle.

IBS-SSS was used to assess clinical improvement of IBS symptoms. A clinical response (≥50-point reduction in IBS-SSS) was observed in 66.7% of patients during GABA-*Melissa officinalis* treatment versus 33.3% with placebo. Furthermore, GABA-*Melissa officinalis* produced a significant reduction in the IBS-SSS total score compared to placebo (*p* = 0.02, Table 3, Figure 3).

GABA-*Melissa officinalis* treatment resulted in a significantly greater improvement in the “Satisfaction with bowel habits” item compared with placebo (*p* = 0.020). A reduction in “Abdominal pain intensity”, “Number of days with abdominal distention”, and “Interference with daily life” were numerically greater with GABA-*Melissa officinalis* but did not reach statistical significance. No relevant carry-over effects were observed.

No significant differences were observed in the weekly number of bowel movements (*p* = 0.289) or BSC (*p* = 0.870).

No significant differences were observed with GABA-*Melissa officinalis* in HAD-A and HAD-D scores compared with placebo (Table 2).

### 3.4. Compliance and Safety Evaluation

Compliance to the intake of study products was generally high, with a median compliance of 96.0 (7.0) %, without any difference between cycle or product.

Monitoring of vital signs indicated no clinically significant values outside normal ranges. Both GABA-*Melissa officinalis* and placebo were generally well tolerated with no serious adverse events or deaths. None of the adverse events required unblinding of the study intervention.

### 3.5. Evaluation of Intestinal Permeability and Systemic Inflammation

**Figure 4:**
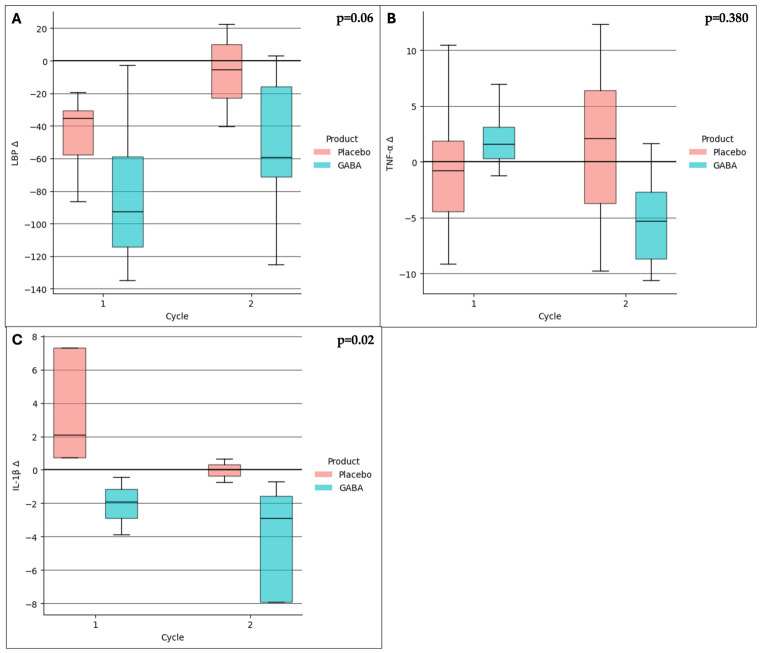
Circulating levels of LBP (panel (**A**)), TNFα (panel (**B**)) and IL-1β (panel (**C**)) in IBS patients according to product and cycle.

Circulating levels of LBP (Figure 4A), TNFα (Figure 4B), and IL-1β (Figure 4C) were compared between the GABA and placebo groups across the two study cycles. IL-1β levels were significantly reduced in the GABA group in comparison with placebo (median change in cycle 1: −1.9 [1.7] vs. 2.9 [6.6]; median change in cycle 2: −2.9 [6.3] vs. 0.0 [0.7]; *p* = 0.02, Figure 4C).

LBP levels showed a non-significant trend toward reduction in the GABA group in both cycles (median change in cycle 1: −92.4 [55.3] vs. −35.3 [27.0]; median change in cycle 2: −59.4 [55.2] vs. −5.6 [33.2]; *p* = 0.06, Figure 4A), whereas TNFα levels did not differ between groups, with substantial intra-group variability (Figure 4B).

### 3.6. Fecal Microbiota

#### 3.6.1. Evaluation of Treatment Sequence Effects on the Microbiome

**Figure 5:**
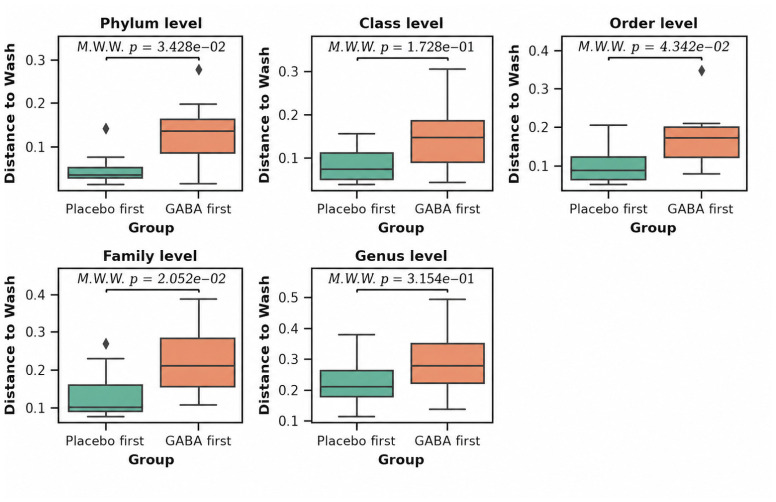
Differences in Bray–Curtis distances (beta-diversity) between baseline and the wash cycle for patients that started the first cycle with the placebo (Placebo first) and those that started with GABA (GABA first). Differences are shown stratified by taxonomic levels. Mann–Whitney U tests were performed.

Taxonomic profiling was conducted using QIIME 2 to characterize the microbial composition across all samples. To evaluate whether beta-diversity patterns differed according to treatment sequence, we compared patients who initiated the first treatment cycle with placebo to those who initiated with GABA (Figure 5).

Beta-diversity analyses revealed significant differences between the two treatment-sequence groups at the phylum, order, and family taxonomic levels (*p* < 0.05; Figure 5). Notably, patients who received GABA in the first cycle exhibited greater beta-diversity distances between the washout and baseline time points compared with the placebo-first group, indicating that GABA administration might have induced microbiome compositional changes that persisted beyond the washout period and might have influenced the microbial community in subsequent cycles.

Given the evidence of a carryover effect from a microbiological point of view, pooling samples across cycles based solely on treatment condition may confound treatment-related inferences. Therefore, all downstream analyses accounted for treatment cycle and sequence effects to appropriately model the longitudinal and crossover structure of the study design.

#### 3.6.2. Alpha and Beta Diversity Changes Between Treatment Groups

Based on the initial beta-diversity analyses, subsequent analyses were performed by jointly accounting for both treatment and cycle. We evaluated both alpha and beta diversity to assess the impact of GABA versus placebo across cycles.

Alpha diversity analyses, encompassing multiple metrics including species richness, Shannon and Simpson diversity, did not reveal significant differences between treatment–cycle combinations at any taxonomic level, from phylum to genus (*p* > 0.05, Figures S1–S5). These results suggest that, although GABA induced persistent shifts in community composition detectable via beta-diversity, overall, within-sample diversity was not significantly altered by treatment or cycle.

On top of that, beta-diversity analyses, performed using both Bray–Curtis and Jaccard distance metrics, did not reveal significant stratification of the overall microbiome composition between treatment–cycle groups at any taxonomic level (from phylum to genus, PERMANOVA, *p* > 0.05; Figures S6–S10) even when doing all pairwise comparisons between treatment groups (Tables S1 and S2). These findings indicate that the overall microbiome composition might not be strongly affected by the treatment (Figure 5).

#### 3.6.3. Microbial Taxa and Functions Differentially Abundant Between Treatment Groups

**Figure 6:**
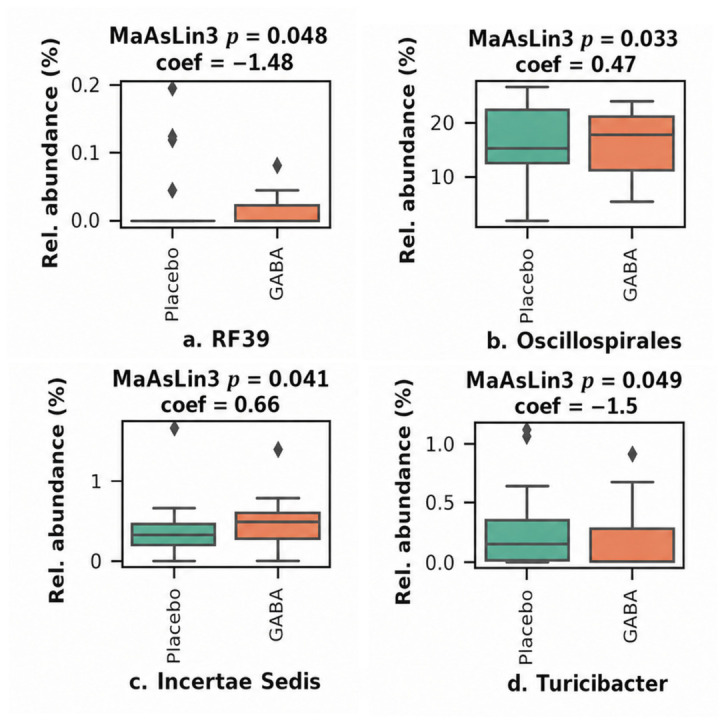
Differences in Bray–Curtis distances (beta-diversity) between baseline and the wash cycle for patients with microbial taxa whose relative abundance was shown significantly different between treatments (*p* < 0.05). Linear models were corrected by sex, age, BMI and abundance at baseline. For abundances at the C3, the wash period was considered as baseline. Negative coefficients are associated with the placebo while positive are associated with GABA.

To identify microbial taxa that exhibited significant changes between treatment groups, we applied MaAsLin3 linear models, adjusting for sex, age, BMI, and baseline abundance. The analysis revealed two microbial taxa associated with the Placebo group, i.e., genus RF39 and genus *Turicibacter*, and two taxa associated with the GABA group, i.e., order *Oscillospirales* and genus *Incertae Sedis* (MaAsLin3 *p* < 0.05; Figure 6). However, none of these associations remained statistically significant after correction for multiple hypothesis testing. While for the *Oscillospirales* order there are no links to GABA production or modulation in the scientific literature, *Incertae Sedis* has been correlated with GABA-related outcomes in animal studies [45].

We further explored the associations between the treatments and microbial functions using a manually curated list of taxa and their corresponding functional links. MaAsLin3 analysis, performed by collapsing the abundances of the taxa associated with each function, did not reveal any statistically significant differences between the treatment groups (MaAsLin3 *p* > 0.05, Figure S11).

### 3.7. Analysis of Tablet Contents

The RP-HPLC analysis of GABA and rosmarinic acid resulted in 505 ± 22 mg/g and 2.19 ± 0.22 mg/g, respectively (Supplementary Information S2 and S3). Since the tablet used in this study weighs on average 552 mg, the resulting amount of GABA and rosmarinic acid would then result in 279 mg/tablet and 1.2 mg/tablet, respectively. These results are coherent with the nominal amount declared, which is 250 mg GABA and 50 mg *M. officinalis* (2% rosmarinic acid) [46].

## 4. Discussion

In this randomized, double-blind, placebo-controlled crossover pilot trial, we investigated the clinical and biological effects of a GABA-*Melissa officinalis* supplementation in patients with IBS-D. The primary endpoint was an improvement in quality of life of these patients, measured through the multiparametric SF-36 questionnaire. The study was powered based on the SF-36 Bodily Pain domain, selected as a clinically relevant and potentially responsive component of the questionnaire in this patient population. Overall, a four-week administration of the tested product did not result in a clinically meaningful improvement in the primary endpoint, as no significant improvement was observed in the composite scores of the SF-36 or in the selected subdomain of the score (Bodily Pain). However, a statistically significant improvement was observed in the “Role Limitations—Emotional” domain during GABA-*Melissa officinalis* supplementation compared with placebo, suggesting a potential beneficial effect on the emotional impact of IBS-D. However, this finding should be interpreted cautiously given the absence of consistent effects across other SF-36 domains.

Although quality of life was the primary endpoint, the most consistent signal in this pilot study emerged from the assessment of symptom severity. Indeed, GABA-*Melissa officinalis* treatment was associated with a significant reduction in the IBS-SSS total score compared with placebo (*p* = 0.02) with 66.7% of patients achieving a clinical response (≥50-point reduction) versus 33.3% during placebo exposure. These findings, as secondary endpoints, should be considered exploratory and hypothesis-generating.

The magnitude of change in IBS-SSS total score with GABA-*Melissa officinalis* treatment (median reduction of −118.5 points during cycle 1 and −56.5 points during cycle 2) exceeds the threshold considered clinically relevant and suggests a substantial symptomatic benefit of this treatment (although this effect should be interpreted in light of the study design and sample size limitation), contrasting what was observed with placebo (median reduction of −36.0 points during cycle 1 and −27.0 points during cycle 2). Even though the reduction was generally more marked with GABA-*Melissa officinalis* treatment compared to placebo, no significant changes were observed in stool frequency or consistency, indicating that the clinical improvement was not primarily driven by changes in bowel habit per se, but rather by global symptom perception, abdominal pain and bowel satisfaction. Taken together, our findings suggest that a four-week GABA supplementation exerts a clinically meaningful effect on global symptom severity in IBS-D patients, with partial but detectable impact on quality of life. The improvement in IBS-SSS, without clinically significant changes in bowel frequency or stool consistency parallels observations from studies targeting neuromodulatory pathways or using agents active on GABAergic system, where sensory modulation may be more pronounced than motor effects [47,48,49]. Furthermore, the observed improvement in the emotional limitation domain of SF-36 may reflect the modulation of the gut–brain axis signaling, although HADS scores did not significantly change. This last aspect may be related to relatively low HADS scores at baseline for many patients, together with the small sample size.

It is still debated how GABA exerts its beneficial effects in IBS-D patients and clinical evidence supporting the use of GABAergic agents in IBS-D remains limited [18]. However, a strong biological rationale exists, as GABAergic signaling regulates visceral nociception, intestinal motility, epithelial barrier integrity and immune response within the gut–brain axis [15,16,18]. Altered GABA receptors expression and dysregulation GABA metabolism have been described in IBS-D patients, suggesting impaired inhibitory control mechanisms [22]. Evidence from preclinical models supports a link between visceral hypersensitivity, inflammation and intestinal barrier disruption mechanisms that contribute to chronic abdominal pain in IBS [23]. In this respect, molecules capable of modulating immune-inflammatory signaling have been shown to influence visceral nociception [20,50]. Thus, the therapeutic role of GABA modulation in IBS-D is currently supported primarily by mechanistic and preclinical evidence, with limited clinical confirmation [18]. The results from our pilot study extended these observations to a clinical IBS-D population, showing not only symptom reduction but also biologically coherent changes in LBP and pro-inflammatory cytokines.

One of the strengths of this study lies in the integration of clinical outcomes with biological markers related to epithelial barrier integrity, systemic inflammation and microbiota analysis, to better understand the possible action of GABA supplementation on IBS symptoms. *Melissa officinalis* has shown to prevent GABA metabolism in in vivo studies through the inhibition of GABA transaminase (GABA-T) and therefore may enhance the therapeutic effects of GABA, extending its half-life and bond to specific GABA receptors [23].

IBS-D is frequently associated with increased intestinal permeability and low-grade inflammation/immune activation, both of which are believed to contribute to visceral hypersensitivity and symptom persistence [1,10,12,13]. GABA treatment was associated with a significant reduction in circulating IL-1β levels compared to placebo (*p* = 0.02), whereas LBP showed a non-significant trend toward reduction (*p* = 0.06). In contrast, TNFα levels did not show appreciable changes and were characterized by high intra-group variability.

These results only partially confirm previous observations by Lucarini et al. [23] which concluded that GABA-Melissa officinalis administration was able to reduce visceral pain and intestinal inflammation in a rat model of post-inflammatory IBS; the improvement was associated with a reinforcement of intestinal barrier integrity [23].

The observed reduction in IL-1β supports the hypothesis that GABA may attenuate inflammatory signaling pathways involved in IBS-D pathophysiology. LBP showed a non-significant trend toward reduction, suggesting a potential effect on intestinal barrier integrity. However, these exploratory findings should be interpreted with caution given the limited sample size.

Mechanistically, GABA has been reported to modulate cytokine production and support intestinal epithelial barrier function, potentially contributing to reduced mucosal inflammation [23,51]. Furthermore, these findings are mechanistically coherent with preclinical data showing that GABAergic signaling modulates immune cell activation, reduces NF-κB pathway activation, and supports tight junction integrity [15,20,23]. Nonetheless, the variability observed in this study highlights the need for larger trials to confirm the consistency and magnitude of these effects. Moreover, the fact that stool frequency and consistency did not significantly change, reinforces the hypothesis that the benefit was not primarily secretory or motility-driven, but more probably related to sensory and barrier mechanisms.

Overall, these results suggest that interventions targeting barrier dysfunction and inflammation may represent promising therapeutic strategy in IBS-D. While these mechanisms may contribute to the improvement of clinical outcomes, the modest and only partially significant changes observed in this study warrant cautious interpretation and require confirmation in larger cohorts.

In this randomized crossover study, we also investigated the effects of GABA treatment on gut microbiome composition, to understand if the effects of GABA supplementation may act through a microbiota-dependent mechanism. The study design, which included two treatment cycles separated by a washout period, allowed us to evaluate treatment-specific effects while accounting for inter-individual variability and potential carryover effects.

The microbiota analysis provided additional insights. Initial analyses revealed significant treatment-sequence effects, with patients receiving GABA in the first cycle exhibiting greater beta-diversity distances between baseline and washout, suggesting that GABA may induce persistent changes in microbial composition. Despite these observations, alpha diversity metrics did not differ significantly between treatment–cycle groups at any taxonomic level, and beta-diversity analyses revealed no clear stratification of overall microbiome composition. At the taxonomic level, two microbial taxa were found associated with GABA treatment, i.e., order *Oscillospirales* and genus *Incertae Sedis*, the latter of which has been previously correlated with GABA-related outcomes in the literature [43,52]. However, these associations did not pass multiple hypothesis testing. Analyses of potential functional impacts, based on a manually curated list of taxa and their associated functions, did not reveal statistically significant differences between treatment groups. Therefore, no substantial microbiota remodeling was observed (no significant alpha/beta diversity changes; taxa associations failed multiple testing correction), likely due to the small sample and short-term intervention.

Overall, our exploratory findings suggest that short-term GABA supplementation may lead to modest, taxon-specific shifts in the gut microbiome but does not result in substantial or reproducible changes in overall microbial diversity or functional potential. Thus, despite the clinical efficacy and possible biological modulation of permeability and inflammatory markers, the overall microbiome structure remained largely stable, suggesting that the clinical effect of GABA in this study likely stems from direct GABAergic modulation of barrier/neuroimmune pathways, rather than microbiota remodeling.

While a subtle taxon-specific effect cannot be excluded, especially given the small sample size and 16S-based resolution, our findings suggest that barrier modulation and immune regulation may represent more relevant therapeutic targets than microbiota reshaping in this context.

### Limitations of the Study

Our study has several limitations that must be acknowledged.

First, the small sample size (18 patients completed the study) limits the statistical power and increases the risk of both type I and type II error, particularly for secondary endpoints and microbiota analyses. This limited sample size may have reduced the ability to detect significant differences in the primary endpoint (SF-36 domains or composite scores); therefore, negative findings should also be interpreted with caution.

Furthermore, a sex imbalance between the study arms was observed at baseline. Although randomization was performed appropriately, the small sample size may have contributed to this uneven distribution by chance, and this imbalance may have influenced the observed outcomes. However, the crossover design partially mitigates between-subject variability as each participant serves as their own control. As a pilot study, the findings should be interpreted as exploratory and require confirmation in multicenter randomized clinical trials on a larger sample.

Second, although no clinically relevant carryover effect was detected for symptom outcomes, microbiota analyses suggest potential sequence-related persistent effects. The two-week washout period was estimated based on GABA pharmacokinetics. However, longer washout may be required to fully exclude microbiological carryover, particularly in light of the sequence-dependent differences observed in beta-diversity.

Third, intestinal permeability was indirectly assessed through circulating LBP rather than direct functional tests. The choice of LBP as marker of intestinal permeability was made to mirror the approach used in the preclinical model. However, LBP is an indirect surrogate marker and does not provide a direct functional assessment of intestinal barrier integrity. Further studies should focus on direct intestinal barrier evaluation to confirm these exploratory results.

Fourth, inflammatory markers were limited to systemic cytokines and may not fully reflect mucosal immune activation, as their variations may be related to multiple extraintestinal causes.

Regarding the microbiota assessment, it was performed using 16S rRNA sequencing, which is recognized as a valid option in exploratory settings but does not provide strain-level resolution nor direct functional metagenomic profiling, limiting the mechanistic interpretation of the observed microbial changes. Furthermore, the current study may have been underpowered to detect subtle microbiota changes, necessitating validation in larger cohorts.

Finally, the monocentric design and relatively young cohort may limit generalizability to older or more heterogeneous IBS-D population. However, this aspect may reflect the higher prevalence of this disease in the younger population [3].

Despite these limitations, exploratory evidence from this pilot study showed a possible improvement in IBS-SSS total score following GABA–*Melissa officinalis* administration, together with a reduction in a LBP and some pro-inflammatory cytokines this suggests that this supplementation may represent a promising therapeutic strategy for the management of abdominal pain in IBS-D. On the other hand, short-term administration of GABA–*Melissa officinalis* did not result in a clinically meaningful improvement in QoL, apart from some effects on daily functioning through improvement in emotional wellbeing. Future larger randomized clinical trials are warranted to confirm our results.

## 5. Conclusions

In this randomized crossover pilot study, four-week GABA-*Melissa officinalis* supplementation may produce a significant improvement in global IBS symptom severity and in emotional QoL domain in IBS-D patients. GABA administration also led to a decrease in circulating LBP and IL-1β levels compared to placebo, although only the reduction in IL-1β reached statistical significance. In contrast, no substantial remodeling of the gut microbiota was observed, indicating that the therapeutic effect of GABA may be largely independent of broad microbiota shifts and more closely related to barrier-neuroimmune mechanisms. These exploratory findings from our pilot study provide preliminary clinical evidence supporting a possible role of GABAergic modulation as a biologically plausible strategy to treat visceral hypersensitivity in IBS-D, requiring confirmation in adequately powered randomized clinical trials.

## Figures and Tables

**Table 1:** Patients’ baseline demographic features.

		Group A Placebo–GABA (*n* = 10)	Group B GABA–Placebo (*n* = 8)	*p*-Value
Demographic features	Sex M:F, *n* (%)	1 (10.0%): 9 (90.0%)	7 (87.5%): 1 (12.5%)	0.003 ^1^
	Age (years), median (IQR)	24.5 (7.75)	28.5 (14.3)	0.624 ^2^
	BMI, median (IQR)	22.4 (6.3)	22.3 (8.2)	1.000 ^2^
Abdominal and fecal symptoms	Weekly bowel movements, median (IQR)	21.5 (12.3)	15.5 (7.75)	0.397 ^2^
	Stool consistency (BSC), median (IQR)	6.0 (0.8)	6.0 (0.0)	0.457 ^2^
	IBS-SSS total, median (IQR)	310.0 (134.0)	345.0 (89.0)	1.000 ^2^
SF-36 (QoL)	SF-36 Physical functioning, median (IQR)	95.0 (8.8)	100.0 (1.3)	0.103 ^2^
	SF-36 Role Limitations—Physical Health, median (IQR)	50.0 (18.8)	62.5 (37.5)	0.886 ^2^
	SF-36 Bodily Pain, median (IQR)	55.0 (20.0)	56.3 (16.9)	0.858 ^2^
	SF-36 General Health, median (IQR)	47.5 (18.8)	65.0 (21.3)	0.118 ^2^
	SF-36 Vitality, median (IQR)	40.0 (10.0)	37.5 (21.3)	1.000 ^2^
	SF-36 Social Functioning, median (IQR)	50.0 (21.9)	50.0 (18.8)	0.715 ^2^
	SF-36 Role Limitations—Emotional Problems, median (IQR)	33.3 (66.7)	50.0 (75.0)	1.000 ^2^
	SF-36 Mental Health, median (IQR)	60.0 (14.0)	60.0 (15.0)	0.892 ^2^
	PCS, median (IQR)	52.0 (1.9)	52.5 (1.6)	0.286 ^2^
	MCS, median (IQR)	48.5 (3.2)	48.7 (3.6)	0.897 ^2^
HADS (psychological)	HAD-A, median (IQR)	11.5 (8.5)	6.5 (4.3)	0.350 ^2^
	HAD-D, median (IQR)	5.5 (5.0)	5.0 (3.3)	0.447 ^2^

^1^ Fisher’s Exact Test; ^2^ Mann–Whitney’s U test. BMI: body mass index; F: female; GABA: γ-aminobutyric acid; IBS-D: diarrhea-predominant irritable bowel syndrome; IQR: interquartile range; HADS: hospital anxiety and depression scale; HAD-A: HADS subdomain anxiety; HAD-D: HADS subdomain depression; M: male; MCS, mental composite score; PCS, physical composite score; QoL: quality of life; SF-36: Short-Form 36.

**Table 2:** SF-36 and HADS scores according to products and cycles.

SF-36 Item	Cycle	GABA		Placebo		*p*-Value ^1^
		*n*	Median (IQR)	*n*	Median (IQR)	
Physical functioning Δ	1	8	0.0 (3.8)	10	0.0 (2.5)	0.638
	2	10	0.0 (1.3)	8	0.0 (0.0)	
Role Limitations—Physical Δ	1	8	12.5 (0.0)	10	−12.5 (31.2)	0.193
	2	10	25.0 (37.5)	8	25.0 (25.0)	
Role Limitations—Emotional Δ	1	8	16.7 (83.3)	10	0.0 (0.0)	0.009
	2	10	0.0 (41.7)	8	0.0 (91.7)	
Vitality Δ	1	8	15.0 (17.5)	10	2.5 (17.5)	0.576
	2	10	−7.5 (22.5)	8	0.0 (20.0)	
Mental Health Δ	1	8	12.0 (16.0)	10	8.0 (19.0)	0.698
	2	10	6.0 (21.0)	8	0.0 (15.0)	
Social functioning Δ	1	8	6.3 (12.5)	10	0.0 (33.8)	0.67
	2	10	6.3 (28.1)	8	6.3 (21.9)	
Bodily pain Δ	1	8	0.0 (21.9)	10	0.0 (24.4)	0.14
	2	10	11.3 (25.0)	8	1.3 (30.0)	
General Health Δ	1	8	0.0 (26.3)	10	7.5 (21.3)	0.07
	2	10	0.0 (6.3)	8	2.5 (5.0)	
PCS Δ	1	8	0.3 (1.2)	10	−0.6 (1.0)	0.105
	2	10	−0.1 (1.8)	8	0.1 (1.8)	
MCS Δ	1	8	1.3 (4.7)	10	0.4 (1.7)	0.978
	2	10	0.8 (3.1)	8	0.1 (4.4)	
HADS	Cycle	GABA		Placebo		*p*-value ^1^
		*n*	Median (IQR)	*n*	Median (IQR)	
HAD-A Δ	1	8	−1.0 (3.8)	10	−3.5 (6.25)	0.912
	2	10	−1.5 (2.8)	8	0.5 (3.8)	
HAD-D Δ	1	8	−0.5 (4.5)	10	−1.0 (3.25)	0.500
	2	10	0.0 (3.8)	8	0.5 (5.3)	

^1^ *p*-values correspond to the treatment effect estimated from the crossover ART-ANOVA model. GABA: γ-aminobutyric acid; IQR: interquartile range; HADS: hospital anxiety and depression scale; HAD-A: HADS subdomain-anxiety; HAD-D: HADS subdomain, depression; MCS, mental composite score; PCS, physical composite score; SF-36: Short-Form 36.

**Table 3:** Gastrointestinal symptoms according to products and cycles.

Symptoms	Cycle	GABA		Placebo		*p*-Value ^1^
		*n*	Median (IQR)	*n*	Mediana (IQR)	
IBS-SSS Item 1 Abdominal Pain Δ	1	8	−38.0 (72.3)	10	−9.50 (27.8)	0.138
	2	10	−7.5 (22.3)	8	−11.5 (17.0)	
IBS-SSS Item 2 Days with abdominal pain Δ	1	8	−20.0 (42.5)	10	−10.0 (32.5)	0.179
	2	10	−15.0 (17.5)	8	0.0 (17.5)	
IBS-SSS Item 3 Abdominal distention Δ	1	8	−28.5 (39.8)	10	−5.0 (33.8)	0.145
	2	10	−9.5 (27.5)	8	−5.0 (22.8)	
IBS-SSS Item 4 Satisfaction bowel habits Δ	1	8	−7.5 (29.5)	10	−7.5 (17.8)	0.02
	2	10	−30.5 (25.8)	8	4.0 (23.0)	
IBS-SSS Item 5 Bowel interference Δ	1	8	−8.0 (22.5)	10	0.5 (29.3)	0.188
	2	10	−6.5 (33.8)	8	−10.5 (19.5)	
IBS-SSS total score Δ	1	8	−118.5 (122.0)	10	−36.0 (74.5)	0.02
	2	10	−56.5 (107.5)	8	−27.0 (84.0)	
Bowel movements/Week Δ	1	8	−1.5 (7.3)	10	−4 (11.3)	0.289
	2	10	−2.0 (3.25)	8	−1.5 (8.8)	
BSC Δ	1	8	−0.3 (1.2)	10	−0.4 (1.6)	0.870
	2	10	−0.6 (1.1)	8	−0.4 (2.4)	

^1^ *p*-values correspond to the treatment effect estimated from the crossover ART-ANOVA model. BSC: Bristol stool chart; GABA: γ-aminobutyric acid; IQR: interquartile range; IBS-SSS: IBS symptom severity score.

## Data Availability

The data presented in this study are available on request from the corresponding author (the data are not publicly available due ethical restrictions).

## Supplementary Materials

The following supporting information can be downloaded at: https://www.mdpi.com/article/10.3390/nu18101569/s1, Study product RP-HPLC chromatograms and test report (Supplementary Informations S1 and S2); Figure S1: Pairwise comparisons of alpha diversity (based phylum-level taxonomic composition) across treatment groups; Figure S2: Pairwise comparisons of alpha diversity (based class-level taxonomic composition) across treatment groups; Figure S3: Pairwise comparisons of alpha diversity (based order-level taxonomic composition) across treatment groups.; Figure S4: Pairwise comparisons of alpha diversity (based family-level taxonomic composition) across treatment groups; Figure S5: Pairwise comparisons of alpha diversity (based genus-level taxonomic composition) across treatment groups; Figure S6: Multidimensional scaling (MDS) based on beta diversity for phylum-level taxonomic composition; Figure S7: Multidimensional scaling (MDS) based on beta diversity for class-level taxonomic composition; Figure S8: Multidimensional scaling (MDS) based on beta diversity for order-level taxonomic composition; Figure S9: Multidimensional scaling (MDS) based on beta diversity for family-level taxonomic composition; Figure S10: Multidimensional scaling (MDS) based on beta diversity for genus-level taxonomic composition; Figure S11: Differences between treatments in the relative abundance of manually curated microbial functions; Table S1: Pairwise beta-diversity comparison based on Bray–Curtis distances between treatment groups at different taxonomic levels; Table S2: Pairwise beta-diversity comparison based on Jaccard distances between treatment groups at different taxonomic levels.

## Abbreviations

The following abbreviations are used in this manuscript:

ANOVA	Analysis of variance
ART	Aligned Rank Transform
BMI	Body mass index
BSC	Bristol stool chart
F	Female
GABA	γ-aminobutyric acid
HAD-A	Anxiety subscale of HADS
HAD-D	Depression subscale of HADS
HADS	Hospital anxiety and depression scale
IBS	Irritable bowel syndrome
IBS-C	IBS patients with constipation
IBS-D	IBS patients with diarrhea
IBS-M	IBS patients with mixed stool type
IBS-SSS	IBS Symptom Severity Score
IBS-U	IBS patients unclassified
IL-1β	Interleukin-1β
IQR	Interquartile range
LBP	Lipopolysaccharide-binding protein
M	Male
MCS	Mental composite score
MDS	Multidimensional scaling
PCS	Physical composite score
QoL	Quality of life
SEM	Standard error of the mean
SF-36	Short-Form Health Survey-36
TNF-α	Tumor Necrosis Factor-α
