# Supplementary material for: Effects of γ-Aminobutyric Acid (GABA) Supplementation on Symptoms, Quality of Life, Intestinal Permeability, Systemic Inflammation and Gut Microbiota in Patients with IBS-D: A Randomized, Double Blind, Placebo-Controlled, Crossover Pilot Study
Source: Nutrients. 2026 May 14;18(10):1569. doi: 10.3390/nu18101569 (PMC13209747; doi:10.3390/nu18101569)
Supplement: Supplementary file 1 [file nutrients-18-01569-s001.zip › Supplementary figures.pdf]

Supplementary materials

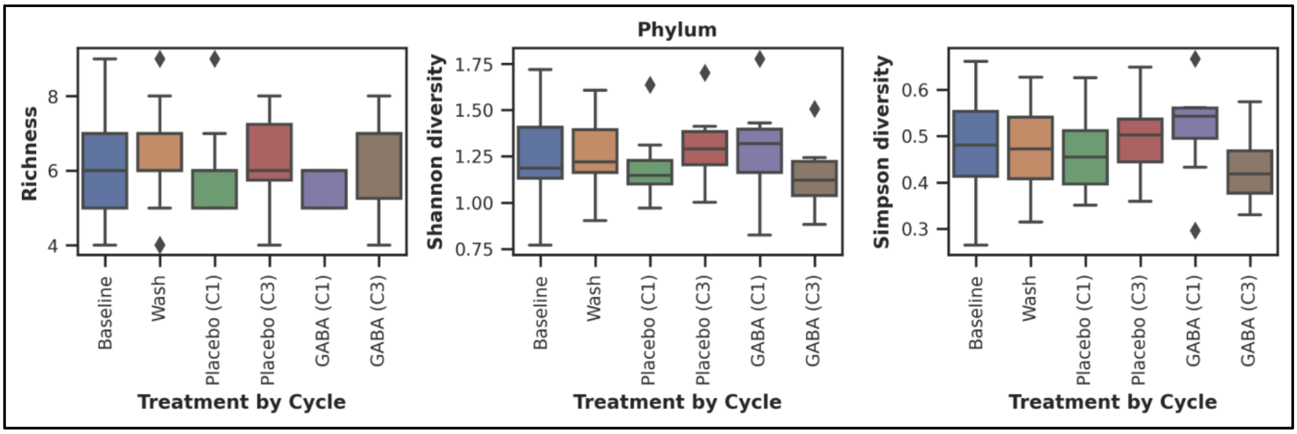

Figure S1. Pairwise comparisons of alpha diversity (based phylum-level taxonomic composition) across treatment groups.
SGB richness, Shannon and Simpson diversity were computed. Significance was assessed using Mann-Whitney U tests (*: 0.01 < p <= 0.05; **: 0.001 < p <= 0.01; ***: 0.0001 < p <= 0.001; ****: p <= 0.0001). Only significant comparisons are shown.

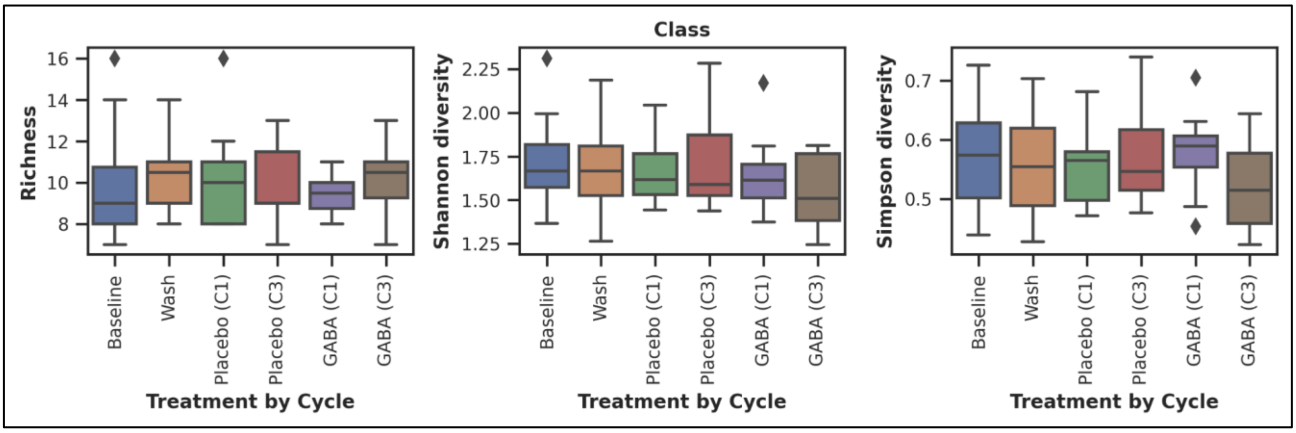

Figure S2. Pairwise comparisons of alpha diversity (based class-level taxonomic composition) across treatment groups.
SGB richness, Shannon and Simpson diversity were computed. Significance was assessed using Mann-Whitney U tests (*: 0.01 < p <= 0.05; **: 0.001 < p <= 0.01; ***: 0.0001 < p <= 0.001; ****: p <= 0.0001). Only significant comparisons are shown.

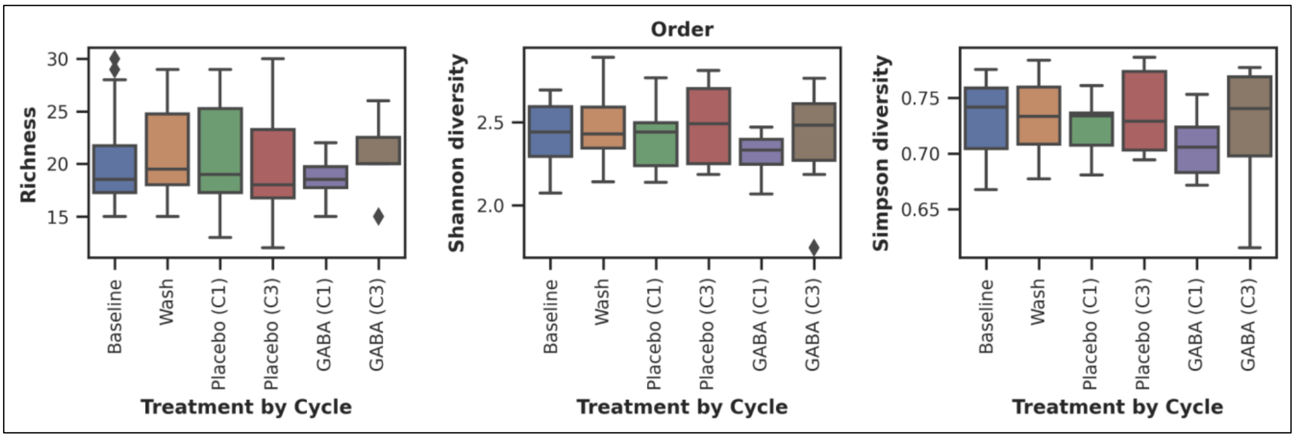

Figure S3. Pairwise comparisons of alpha diversity (based order-level taxonomic composition) across treatment groups.
SGB richness, Shannon and Simpson diversity were computed. Significance was assessed using Mann-Whitney U tests (*: 0.01 < p <= 0.05; **: 0.001 < p <= 0.01; ***: 0.0001 < p <= 0.001; ****: p <= 0.0001). Only significant comparisons are shown.

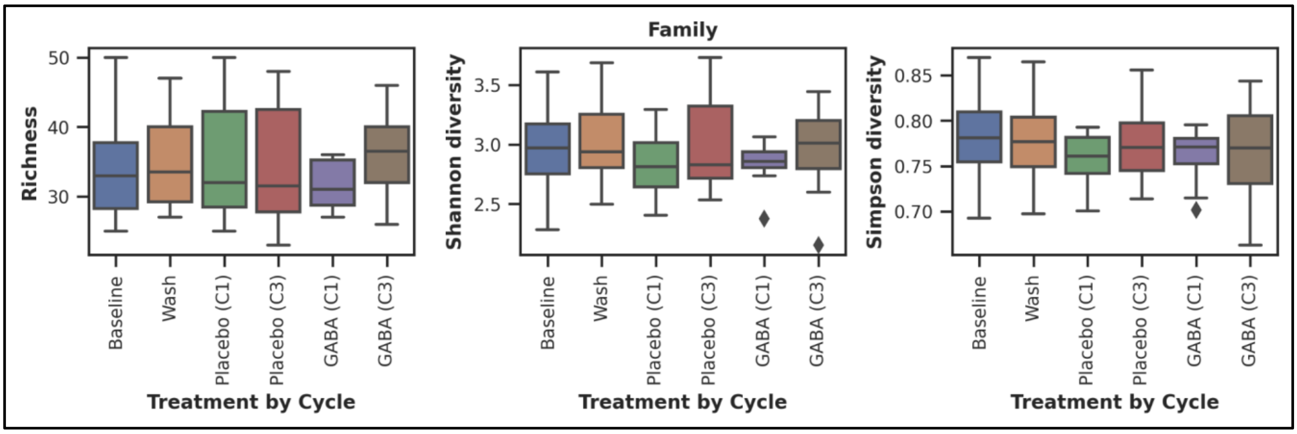

Figure S4. Pairwise comparisons of alpha diversity (based family-level taxonomic composition) across treatment groups.
SGB richness, Shannon and Simpson diversity were computed. Significance was assessed using Mann-Whitney U tests (*: 0.01 < p <= 0.05; **: 0.001 < p <= 0.01; ***: 0.0001 < p <= 0.001; ****: p <= 0.0001). Only significant comparisons are shown.

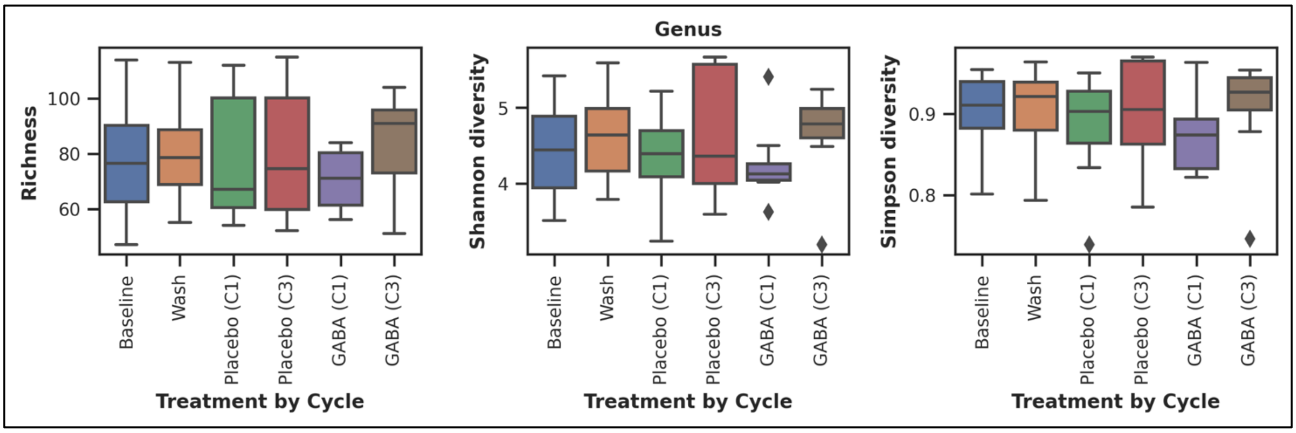

Figure S5. Pairwise comparisons of alpha diversity (based genus-level taxonomic composition) across treatment groups.
SGB richness, Shannon and Simpson diversity were computed. Significance was assessed using Mann-Whitney U tests (*: 0.01 < p <= 0.05; **: 0.001 < p <= 0.01; ***: 0.0001 < p <= 0.001; ****: p <= 0.0001). Only significant comparisons are shown.

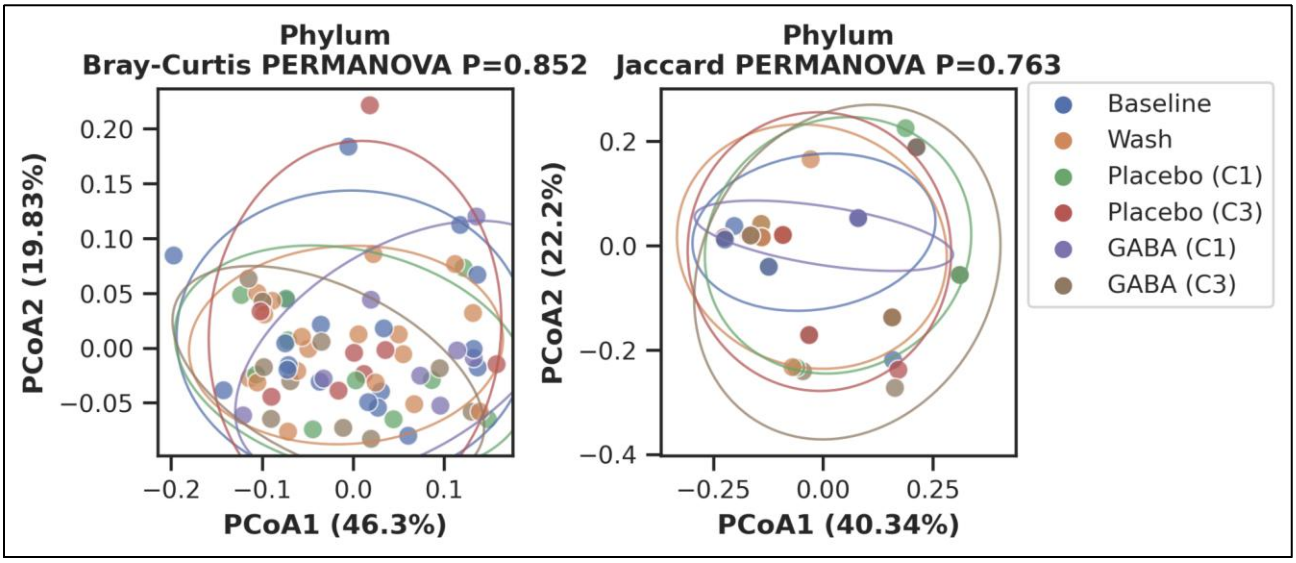

Figure S6. Multidimensional scaling (MDS) based on beta diversity for phylum-level taxonomic composition. Bray-Curtis and Jaccard distances were computed on the arcsine square root-transformed relative abundances. Significant differences were computed using PERMANOVA tests.

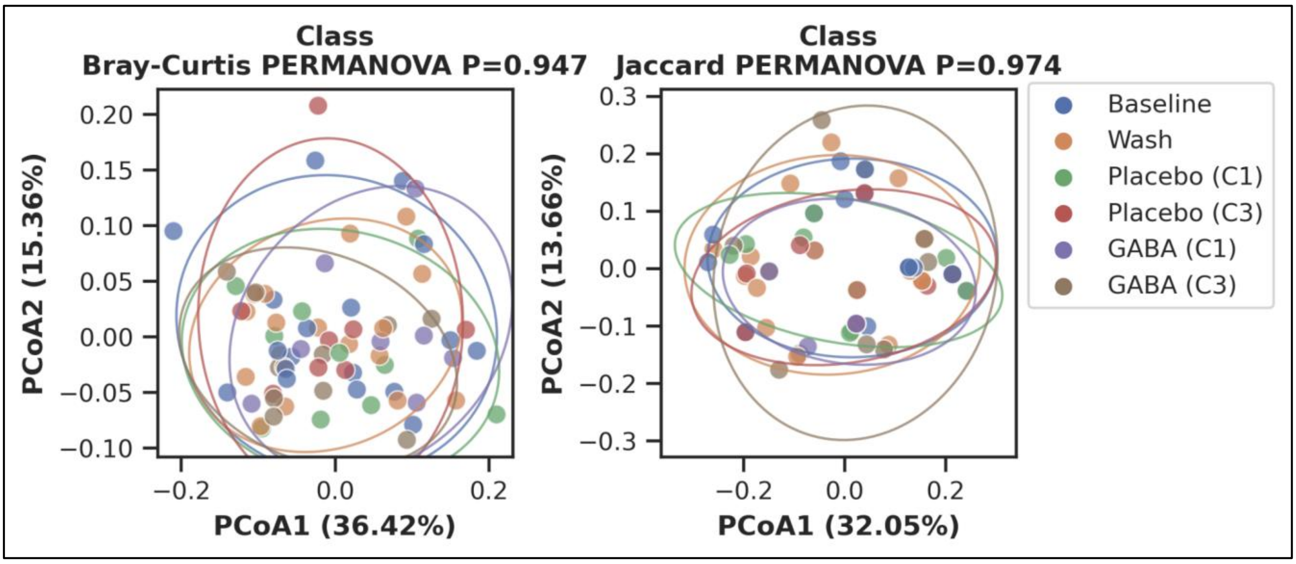

Figure S7. Multidimensional scaling (MDS) based on beta diversity for class-level taxonomic composition. Bray-Curtis and Jaccard distances were computed on the arcsine square root-transformed relative abundances. Significant differences were computed using PERMANOVA tests.

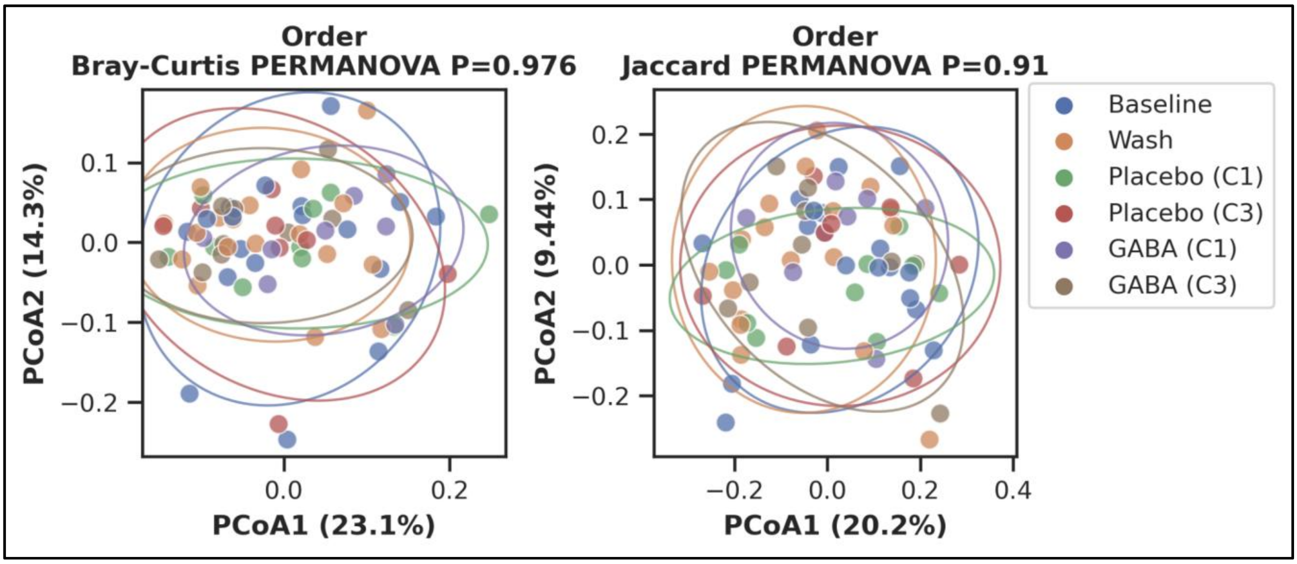

Figure S8. Multidimensional scaling (MDS) based on beta diversity for order-level taxonomic composition. Bray-Curtis and Jaccard distances were computed on the arcsine square root-transformed relative abundances. Significant differences were computed using PERMANOVA tests.

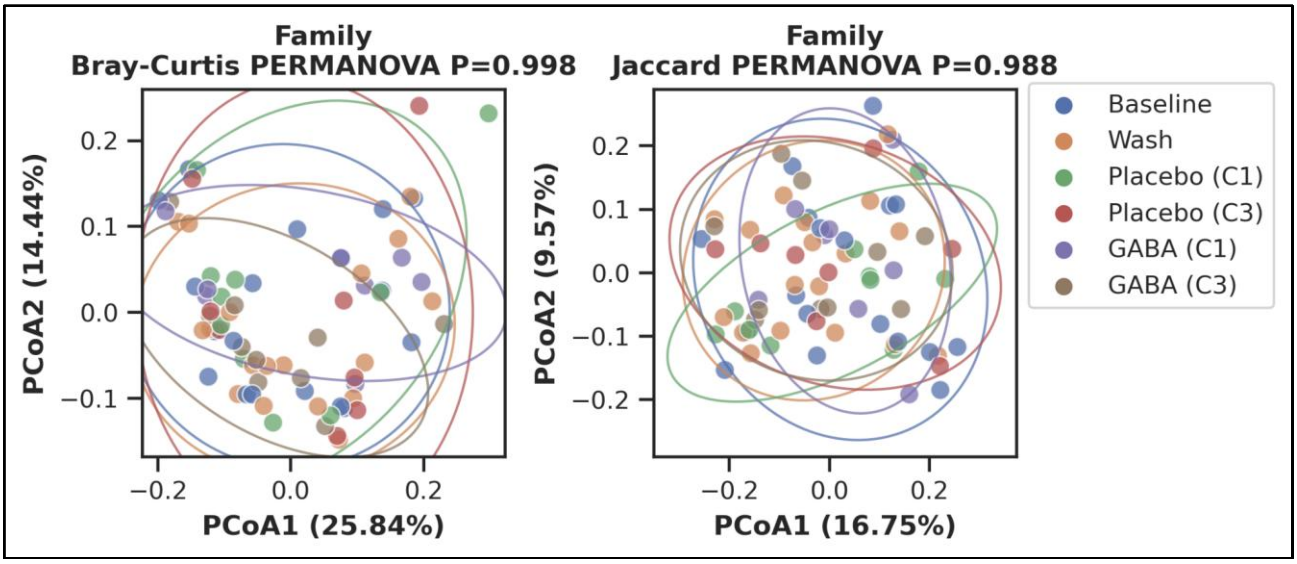

Figure S9. Multidimensional scaling (MDS) based on beta diversity for family-level taxonomic composition. Bray-Curtis and Jaccard distances were computed on the arcsine square root-transformed relative abundances. Significant differences were computed using PERMANOVA tests.

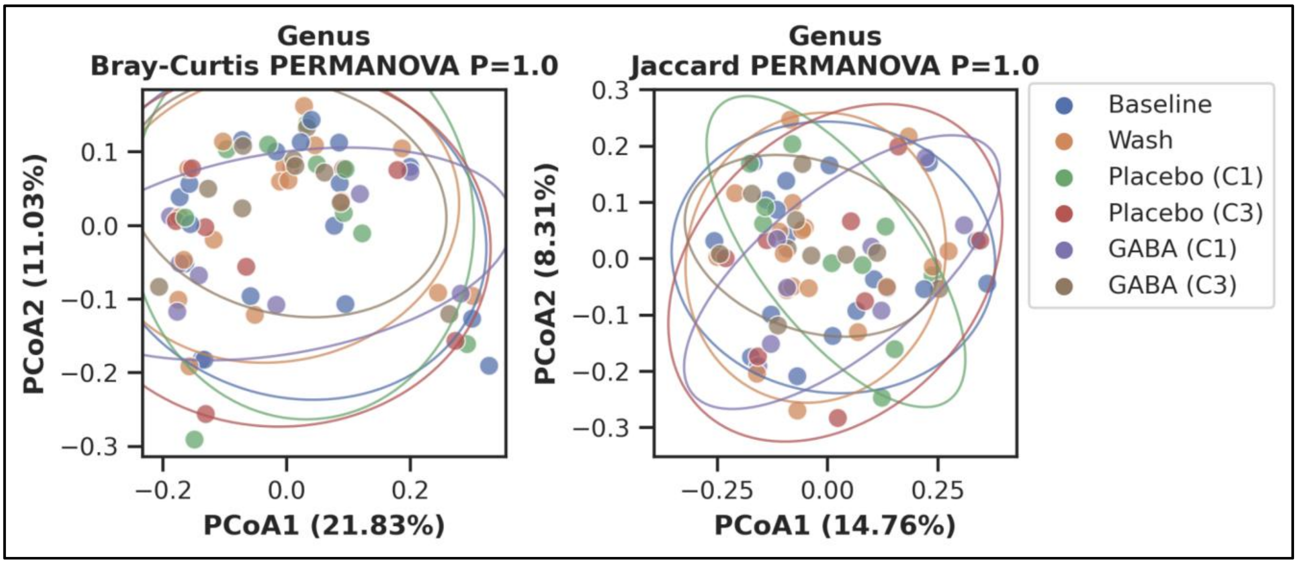

Figure S10. Multidimensional scaling (MDS) based on beta diversity for genus-level taxonomic composition. Bray-Curtis and Jaccard distances were computed on the arcsine square root-transformed relative abundances. Significant differences were computed using PERMANOVA tests.

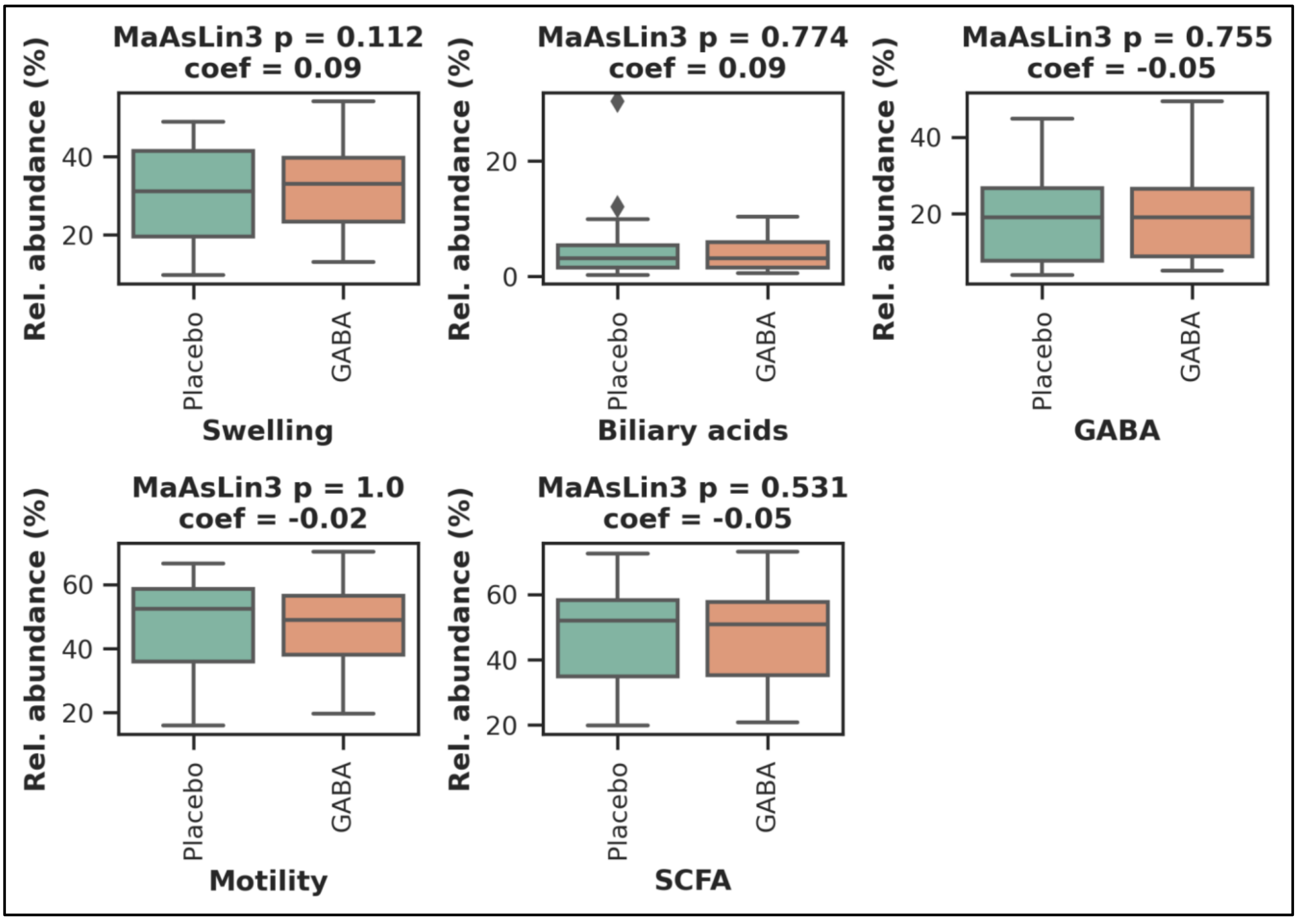

Figure S11. Differences between treatments in the relative abundance of manually curated microbial functions. Linear models were corrected by sex, age, BMI and abundance at baseline. For abundances at the V3, the wash period was considered as baseline. Negative coefficients are associated with the placebo while positive are associated with GABA. No significant associations were found.

Table S1. Pairwise beta-diversity comparison based on Bray-Curtis distances between treatment groups at different taxonomic levels. PERMANOVA p-values are shown per comparison.

**Phylum level**						
	**Baseline**	**Wash**	**Placebo (V1)**	**Placebo (V3)**	**GABA (V1)**	**GABA (V3)**
**Baseline**	0	0.518	0.25	0.701	0.909	0.063
**Wash**	0.518	0	0.611	0.991	0.997	0.274
**Placebo (V1)**	0.25	0.611	0	0.735	0.734	0.546
**Placebo (V3)**	0.701	0.991	0.735	0	0.978	0.546
**GABA (V1)**	0.909	0.997	0.734	0.978	0	0.588
**GABA (V3)**	0.063	0.274	0.546	0.546	0.588	0
**Class level**						
	**Baseline**	**Wash**	**Placebo (V1)**	**Placebo (V3)**	**GABA (V1)**	**GABA (V3)**
**Baseline**	0	0.922	0.937	0.951	0.316	0.507
**Wash**	0.922	0	0.706	0.926	0.143	0.766
**Placebo (V1)**	0.937	0.706	0	0.822	0.348	0.513
**Placebo (V3)**	0.951	0.926	0.822	0	0.291	0.72
**GABA (V1)**	0.316	0.143	0.348	0.291	0	0.089
**GABA (V3)**	0.507	0.766	0.513	0.72	0.089	0
**Order level**						
	**Baseline**	**Wash**	**Placebo (V1)**	**Placebo (V3)**	**GABA (V1)**	**GABA (V3)**
**Baseline**	0	0.968	0.983	0.979	0.409	0.664
**Wash**	0.968	0	0.838	0.94	0.143	0.928
**Placebo (V1)**	0.983	0.838	0	0.891	0.349	0.688
**Placebo (V3)**	0.979	0.94	0.891	0	0.385	0.898
**GABA (V1)**	0.409	0.143	0.349	0.385	0	0.058
**GABA (V3)**	0.664	0.928	0.688	0.898	0.058	0
**Family level**						
	**Baseline**	**Wash**	**Placebo (V1)**	**Placebo (V3)**	**GABA (V1)**	**GABA (V3)**
**Baseline**	0	0.994	0.961	0.935	0.648	0.64
**Wash**	0.994	0	0.773	0.94	0.504	0.706
**Placebo (V1)**	0.961	0.773	0	0.739	0.48	0.874
**Placebo (V3)**	0.935	0.94	0.739	0	0.786	0.675
**GABA (V1)**	0.648	0.504	0.48	0.786	0	0.174
**GABA (V3)**	0.64	0.706	0.874	0.675	0.174	0
**Genus level**						
	**Baseline**	**Wash**	**Placebo (V1)**	**Placebo (V3)**	**GABA (V1)**	**GABA (V3)**
**Baseline**	0	0.999	0.989	0.875	0.685	0.826
**Wash**	0.999	0	0.851	0.911	0.656	0.862
**Placebo (V1)**	0.989	0.851	0	0.609	0.521	0.981
**Placebo (V3)**	0.875	0.911	0.609	0	0.918	0.745
**GABA (V1)**	0.685	0.656	0.521	0.918	0	0.218
**GABA (V3)**	0.826	0.862	0.981	0.745	0.218	0

Table S2. Pairwise beta-diversity comparison based on Jaccard distances between treatment groups at different taxonomic levels. PERMANOVA p-values are shown per comparison.

**Phylum level**						
	**Baseline**	**Wash**	**Placebo (V1)**	**Placebo (V3)**	**GABA (V1)**	**GABA (V3)**
**Baseline**	0	0.907	0.895	0.862	0.244	0.492
**Wash**	0.907	0	0.953	0.761	0.092	0.536
**Placebo (V1)**	0.895	0.953	0	0.774	0.19	0.603
**Placebo (V3)**	0.862	0.761	0.774	0	0.251	0.454
**GABA (V1)**	0.244	0.092	0.19	0.251	0	0.057
**GABA (V3)**	0.492	0.536	0.603	0.454	0.057	0
**Class level**						
	**Baseline**	**Wash**	**Placebo (V1)**	**Placebo (V3)**	**GABA (V1)**	**GABA (V3)**
**Baseline**	0	0.729	0.649	0.925	0.79	0.386
**Wash**	0.729	0	0.936	0.964	0.854	0.524
**Placebo (V1)**	0.649	0.936	0	0.935	0.823	0.731
**Placebo (V3)**	0.925	0.964	0.935	0	0.987	0.807
**GABA (V1)**	0.79	0.854	0.823	0.987	0	0.557
**GABA (V3)**	0.386	0.524	0.731	0.807	0.557	0
**Order level**						
	**Baseline**	**Wash**	**Placebo (V1)**	**Placebo (V3)**	**GABA (V1)**	**GABA (V3)**
**Baseline**	0	0.334	0.969	0.905	0.3	0.493
**Wash**	0.334	0	0.882	0.901	0.25	0.959
**Placebo (V1)**	0.969	0.882	0	0.929	0.467	0.971
**Placebo (V3)**	0.905	0.901	0.929	0	0.815	0.859
**GABA (V1)**	0.3	0.25	0.467	0.815	0	0.17
**GABA (V3)**	0.493	0.959	0.971	0.859	0.17	0
**Family level**						
	**Baseline**	**Wash**	**Placebo (V1)**	**Placebo (V3)**	**GABA (V1)**	**GABA (V3)**
**Baseline**	0	0.752	0.954	0.922	0.756	0.725
**Wash**	0.752	0	0.877	0.895	0.677	0.966
**Placebo (V1)**	0.954	0.877	0	0.814	0.661	0.944
**Placebo (V3)**	0.922	0.895	0.814	0	0.872	0.957
**GABA (V1)**	0.756	0.677	0.661	0.872	0	0.348
**GABA (V3)**	0.725	0.966	0.944	0.957	0.348	0
**Genus level**						
	**Baseline**	**Wash**	**Placebo (V1)**	**Placebo (V3)**	**GABA (V1)**	**GABA (V3)**
**Baseline**	0	0.996	0.986	0.995	0.84	0.967
**Wash**	0.996	0	0.989	0.993	0.87	0.997
**Placebo (V1)**	0.986	0.989	0	0.681	0.467	0.925
**Placebo (V3)**	0.995	0.993	0.681	0	0.982	0.805
**GABA (V1)**	0.84	0.87	0.467	0.982	0	0.137
**GABA (V3)**	0.967	0.997	0.925	0.805	0.137	0
