# Supplementary material for: Effects of γ-Aminobutyric Acid (GABA) Supplementation on Symptoms, Quality of Life, Intestinal Permeability, Systemic Inflammation and Gut Microbiota in Patients with IBS-D: A Randomized, Double Blind, Placebo-Controlled, Crossover Pilot Study
Source: Nutrients. 2026 May 14;18(10):1569. doi: 10.3390/nu18101569 (PMC13209747; doi:10.3390/nu18101569)
Supplement: Supplementary file 1 [file nutrients-18-01569-s001.zip › Supplementary Information S1 – Test report.PDF]

Spettabile/*Dear*:
DEPOFARMA SPA
Via Tintoretto, 9/a
31022 PREGANZIOL (TV)

ID campione:<br>*Sample ID:*	**FD-26-008320-045190**
Identificazione:<br>*Identification:*	**Food supplement with GABA and Lemon Balm - Batch: 196N25 - Expiry: 12/2028**
Data ricezione:<br>*Receiving date:*	27/03/2026
Data rapporto di prova:<br>*Test report date:*	08/04/2026
Matrice:<br>*Matrix:*	Nutraceutici/*Nutraceuticals*
Prelevatore:<br>*Sampler:*	Cliente/Customer
Responsabilità ritiro/trasporto:<br>*Transport responsibility:*	Cliente/*Customer*

**PROVA/*TEST*** **Metodo/*Method***	**U.M.**	**Risultato *Result***	**R**	**Inc. (§) *Unc. (§)***	**Val. Rif. *Ref. Val.***	**Inizio-Fine Prova *Start-End Test***
acido 4-amminobutanoico/*4-aminobutanoic acid*<br>[MI] PPA-365 rev1 2022	mg/g	505		± 22	-	03/04/26-07/04/26
acido rosmarinico/*rosmarinic acid*<br>[MI] PPA-160 rev1 2023	mg/g	2,19	-	± 0,22	-	07/04/26-07/04/26

Le informazioni sottolineate sono fornite dal cliente, il laboratorio ne declina la responsabilità.
U.M. = unità di misura.
R = recupero %. Nel caso di metodi che prevedano fasi di preconcentrazione e purificazione, ove non espressamente indicato, il recupero è da intendersi compreso all'interno dei limiti di accettabilità specifici previsti dal metodo di prova o dalla normativa vigente.
Ove non espressamente indicato il recupero non è stato utilizzato nei calcoli.
Inc. (§) = incertezza estesa associata alla misura con fattore di copertura k=2 e ad un livello di fiducia del 95% per valori quantificati maggiori del LOQ. L'intervallo fiduciario viene espresso indicandone i limiti fiduciari inferiore e superiore separati dal simbolo ÷.
Risultato "<x" = ove non diversamente specificato, indica un valore inferiore al limite di quantificazione del metodo (LOQ).
VAL. RIF. = valore di riferimento.
Le sommatorie di più composti, ove non espressamente indicato, sono state calcolate con il criterio lower bound; LOQ della somma si riferisce al composto meno sensibile.
• = indica il superamento del limite senza considerare l'incertezza di misura.

LA_FOOD_COA_R18.RPT

Questo Rapporto di Prova riguarda solo il campione sottoposto a prova; nel caso in cui il Laboratorio non sia responsabile del campionamento, il Rapporto di Prova riguarda solo il campione sottoposto a prova così come ricevuto.
Il Rapporto di Prova non può essere riprodotto parzialmente salvo approvazione scritta da parte del Responsabile del Laboratorio LabAnalysis Life Science s.r.l.
This Test Report only concerns the tested sample; in the event that the Laboratory is not responsible for sampling, the Test Report only concerns the tested sample as received.
The Test Report cannot be partially reproduced without the written approval by the director of LabAnalysis Life Science s.r.l.

Documento firmato digitalmente secondo la normativa vigente/*Document digitally signed according to current legislation*

The underlined information is provided by the customer, the laboratory declines its responsibility.

U.M. = unit of measure.
R = recovery %. In the case of methods that have preconcentration's and purification's steps, where not expressly indicated, the recovery is to be understood comprised within the specific acceptability limits provided by the test method or by the current legislation. Where recovery is no mentioned it was not used in the calculations.
Unc. (§) = expanded uncertainty associated with the measure with coverage factor k=2 and confidence level of 95% for quantified values greater than the LOQ. The confidence interval is expressed indicating the lower and upper confidence limits separated by the symbol ÷.
Result "<x" = unless otherwise specified, indicates a value lower than the limit of quantification (LOQ).
REF. VAL. = reference value.
The summations of several compounds, where not expressly indicated, were calculated with the lower bound criterion; LOQ of the sum refers to the least sensitive compound.
• = indicates that the limit is exceeded without considering the uncertainty of measurement.

[MI] = analisi eseguite presso il laboratorio di Origgio.*Analysis performed at the Origgio Laboratory*LabAnalysis Life Science s.r.l., Via Saronnino 86/A, 21040, Varese.

Direttore tecnico Laboratorio Chimico/Technical Director
Chemical Laboratory
Ordine Interprovinciale dei Chimici e dei Fisici della Lombardia
Albo Professionale n. 3654/Interprovincial Order of Chemists
and Physicists of Lombardy
Dott.ssa Fausta Giuffrè

Fine rapporto di prova
End of test report

Documento firmato digitalmente secondo la normativa vigente/*Document digitally signed according to current legislation*

Questo Rapporto di Prova riguarda solo il campione sottoposto a prova; nel caso in cui il Laboratorio non sia responsabile del campionamento, il Rapporto di Prova riguarda solo il campione sottoposto a prova così come ricevuto.
Il Rapporto di Prova non può essere riprodotto parzialmente salvo approvazione scritta da parte del Responsabile del Laboratorio LabAnalysis Life Science s.r.l.
This Test Report only concerns the tested sample; in the event that the Laboratory is not responsible for sampling, the Test Report only concerns the tested sample as received.
The Test Report cannot be partially reproduced without the written approval by the director of LabAnalysis Life Science s.r.l.

Sede legale Via Europa, 5 - 27041 Casanova Lonati (PV) |**Tel.** +39 0385 287 128 | info@labanalysis.it | www.labanalysis.it |
LabAnalysis Life Science s.r.l., a socio unico, Società soggetta a direzione e coordinamento da parte di LabAnalysis Group S.r.l. Cap.Soc. €103.000,00 int. vers. Registro Imprese di Pavia - C.F./P.IVA 02235450182 R.E.A. CCIAA di Pavia n. 257033
