# Supplementary material for: Effects of γ-Aminobutyric Acid (GABA) Supplementation on Symptoms, Quality of Life, Intestinal Permeability, Systemic Inflammation and Gut Microbiota in Patients with IBS-D: A Randomized, Double Blind, Placebo-Controlled, Crossover Pilot Study
Source: Nutrients. 2026 May 14;18(10):1569. doi: 10.3390/nu18101569 (PMC13209747; doi:10.3390/nu18101569)
Supplement: Supplementary file 1 [file nutrients-18-01569-s001.zip › Supplementary Information S2 – Chromatograms.pdf]

STD 4-aminobutanoic acid

9 CCV GABA 50 PPM

Sample Name:	CCV GABA 50 PPM	*Injection Volume:*	75,0
Vial Number:	Vial:85	*Channel:*	DAD1_Sign
Sample Type:	Check Standard	*Wavelength:*	n.a.
Control Program:	AA_1	*Bandwidth:*	n.a.
Quantif. Method:	Amminoacidi	*Dilution Factor:*	
Recording Time:	3/4/2026 18:34	*Sample Weight:*	1,0000
Run Time (min):	18,49	*Sample Amount:*	1,0000

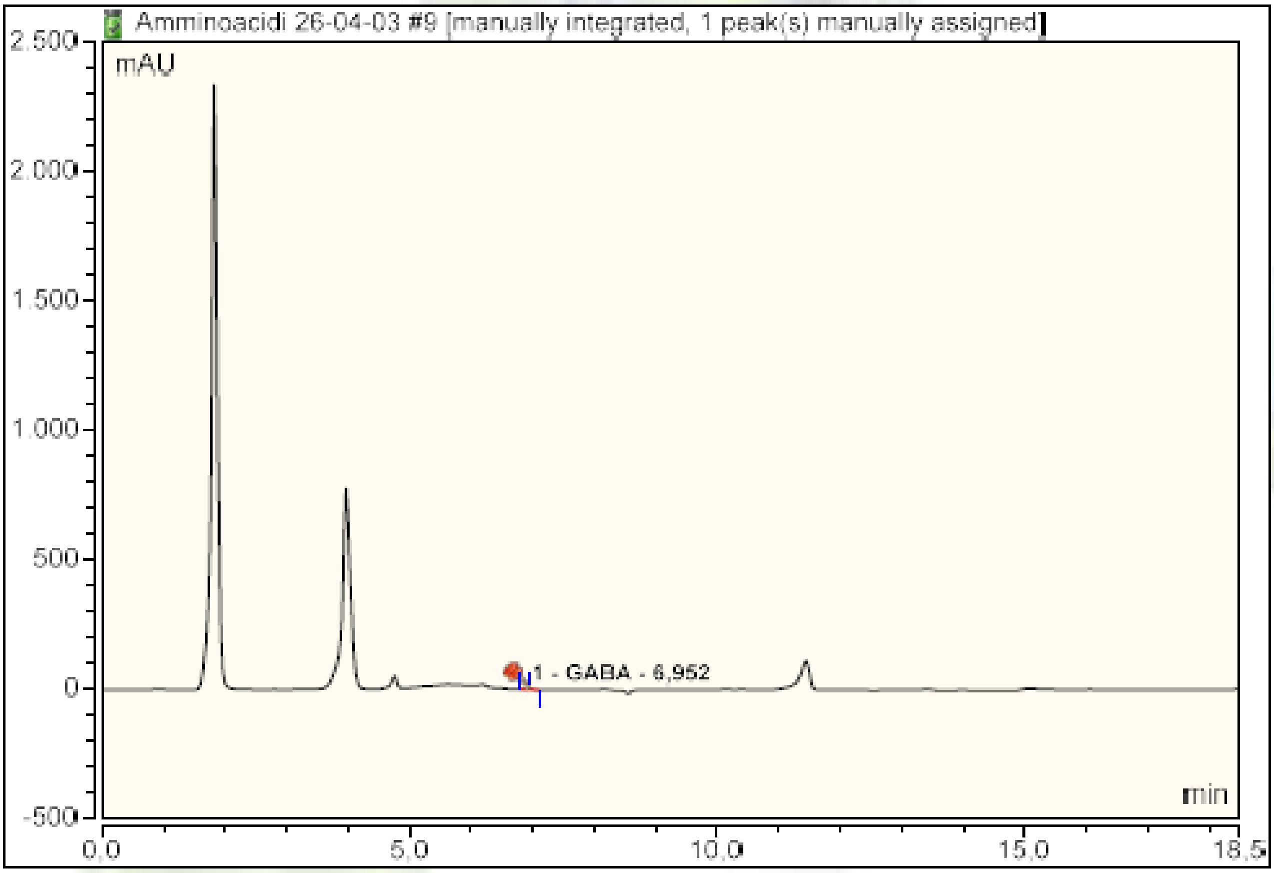

No.	Ret.Time min	Peak Name	Height mAU	Area mAU*min	Rel.Area %	Amount	Type
1	6,95	GABA	5,183582	0,537933	100,00	n.a.	BMB*^
**Total:**			5,184	0,538	100,00	0,000	

Sample FD-26-008320-045190

13 FD-26-008320-045190#DL1 (d20)

Sample Name:	FD-26-008320-045190#DL1 (d20)	*Injection Volume:*	75,0
Vial Number:	Vial:89	*Channel:*	DAD1_Sign
Sample Type:	Unknown	*Wavelength:*	n.a.
Control Program:	AA_1	*Bandwidth:*	n.a.
Quantif. Method:	Amminoacidi	*Dilution Factor:*	
Recording Time:	3/4/2026 19:55	*Sample Weight:*	1,0000
Run Time (min):	18,49	*Sample Amount:*	1,0000

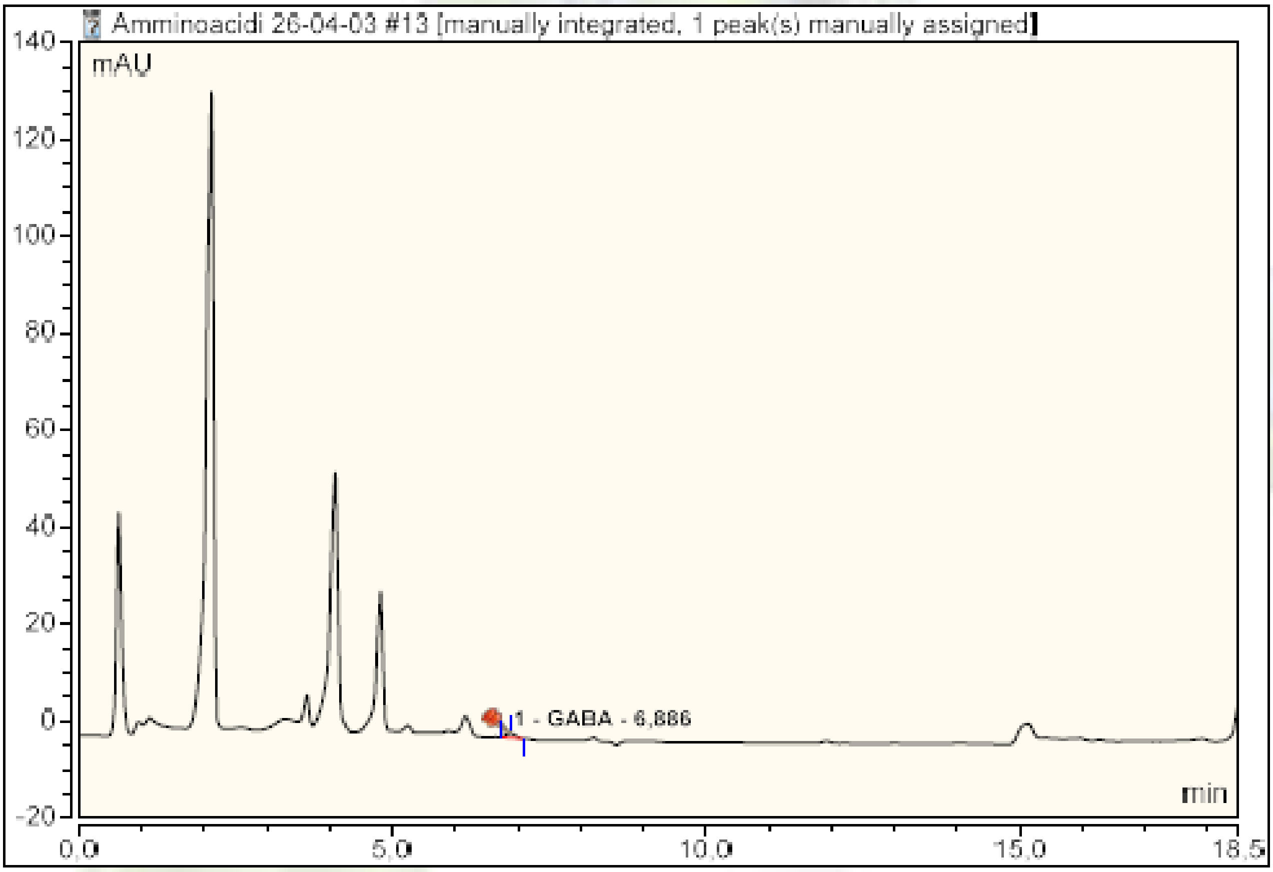

No.	Ret.Time min	Peak Name	Height mAU	Area mAU*min	Rel.Area %	Amount	Type
1	6,89	GABA	0,906028	0,162414	100,00	n.a.	BMB*^
**Total:**			0,906	0,162	100,00	0,000	

STD Rosmarinic acid

Chromatogram and Results

Injection Details

Injection Name:	**CCV-243522**	*Run Time (min):*	**30,00**
Vial Number:	**Y:F9**	*Injection Volume:*	**20,00**
Injection Type:	**Check Standard**	*Channel:*	**UV_VIS_1**
Calibration Level:	**2**	*Wavelength:*	**210**
Instrument Method:	**Verbascoside-Rosmarinico**	*Bandwidth:*	**n.a.**
Processing Method:	**TIMO**	*Dilution Factor:*	**1,0000**
Injection Date/Time:	**07/apr/26 17:15**	*Sample Weight:*	**1,0000**

Chromatogram

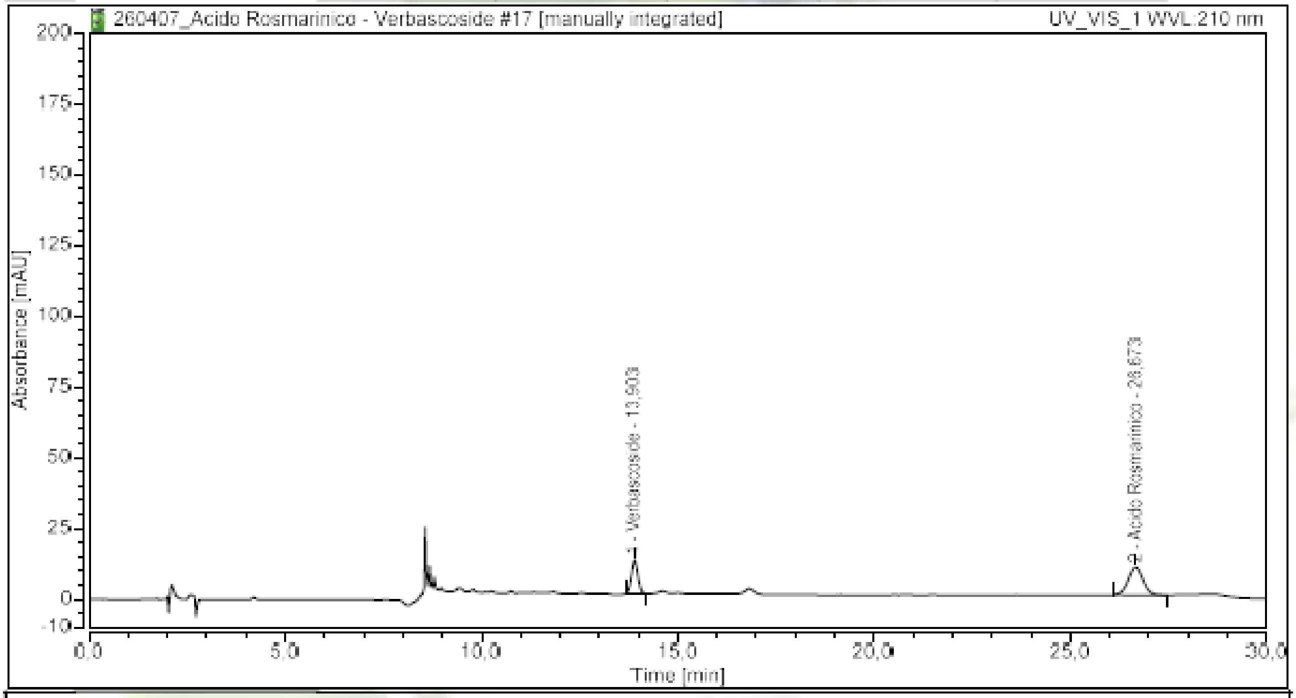

Integration Results

No.	Peak Name	Retention Time min	Area mAU*min	Height mAU	Peak Width (50%) min	Relative Height %	Amount mg/l
1	Verbascoside	13,903	2,214	11,936	0,17	54,52	5,9684
2	Acido Rosmarinico	26,673	4,316	9,955	0,40	45,48	5,3857
**Total:**			**6,530**	**21,891**	**0,57**	**100,00**	

Sample FD-26-008320-045190

Chromatogram and Results

Injection Details

Injection Name:	**FD-26-008320-045190**	*Run Time (min):*	**30,00**
Vial Number:	**Y:F8**	*Injection Volume:*	**20,00**
Injection Type:	**Unknown**	*Channel:*	**UV_VIS_1**
Calibration Level:		*Wavelength:*	**210**
Instrument Method:	**Verbascoside-Rosmarinico**	*Bandwidth:*	**n.a.**
Processing Method:	**TIMO**	*Dilution Factor:*	**1,0000**
Injection Date/Time:	**07/apr/26 16:44**	*Sample Weight:*	**1,0000**

Chromatogram

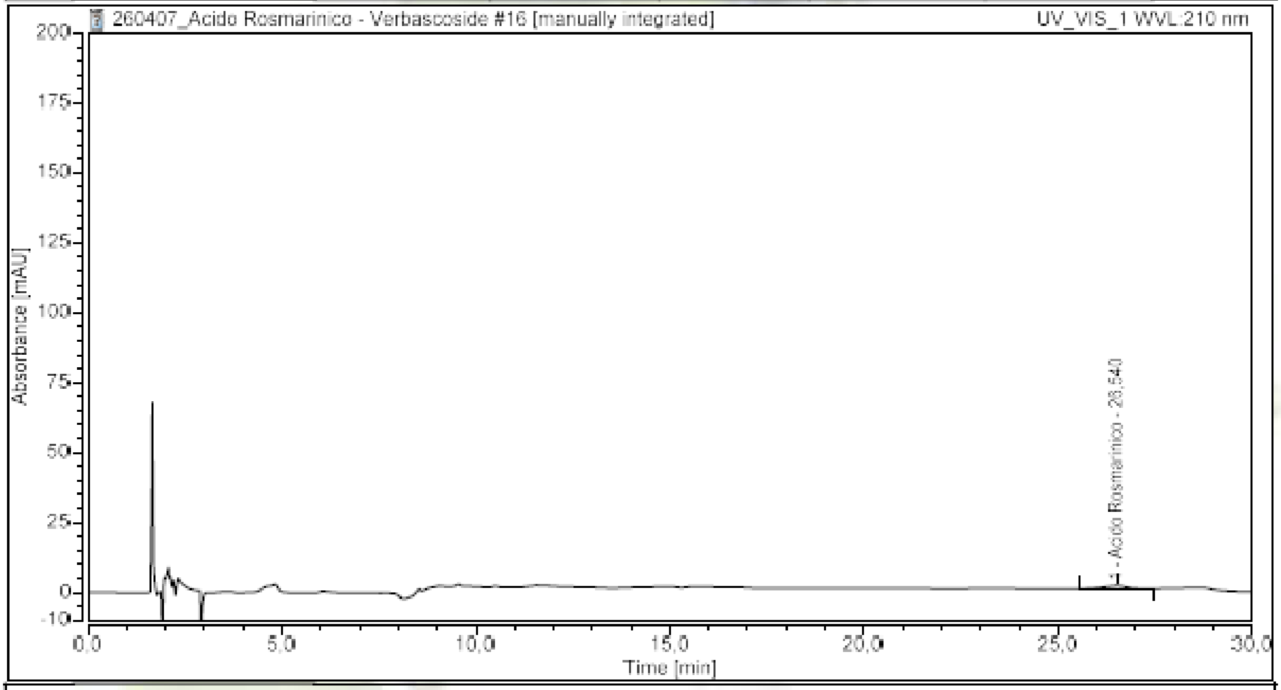

Integration Results

No.	Peak Name	Retention Time min	Area mAU*min	Height mAU	Peak Width (50%) min	Relative Height %	Amount mg/l
n.a.	Verbascoside	n.a.	n.a.	n.a.	n.a.	n.a.	n.a.
1	Acido Rosmarinico	26,540	0,880	1,134	0,70	100,00	1,0982
**Total:**			**0,880**	**1,134**	**0,70**	**100,00**	
